# ﻿A taxonomic revision of GarciniasectionGarcinia (Clusiaceae) in Thailand

**DOI:** 10.3897/phytokeys.244.126207

**Published:** 2024-07-15

**Authors:** Chatchai Ngernsaengsaruay, Pichet Chanton, Nisa Leksungnoen, Minta Chaiprasongsuk, Raweewan Thunthawanich

**Affiliations:** 1 Department of Botany, Faculty of Science, Kasetsart University, Chatuchak, Bangkok 10900, Thailand Kasetsart University Bangkok Thailand; 2 Biodiversity Center, Kasetsart University (BDCKU), Chatuchak, Bangkok 10900, Thailand Suan Luang Rama IX Foundation Bangkok Thailand; 3 Suan Luang Rama IX Foundation, Nong Bon Subdistrict, Prawet District, Bangkok, 10250, Thailand Kasetsart University Bangkok Thailand; 4 Department of Forest Biology, Faculty of Forestry, Kasetsart University, Chatuchak, Bangkok 10900, Thailand Suan Luang Rama IX Foundation Bangkok Thailand

**Keywords:** Agamospermy, dioecy, edible fruits, Guttiferae, Malpighiales, second-step lectotypification, taxonomy

## Abstract

GarciniasectionGarcinia (Clusiaceae) is revised for Thailand with three species and one variety, i.e., two native species: *G.celebica* and *G.exigua*, and one cultivated species: G.mangostanavar.mangostana. Detailed morphological descriptions, illustrations, and an identification key to the species are presented, along with notes on distributions, habitats and ecology, phenology, conservation assessments, etymology, vernacular names, uses, and specimens examined. The section is recognized by its terminal inflorescences of simple cymes, or sometimes a solitary flower; flowers with 4 sepals and 4 petals; male flowers often with a pistillode, and stamens united into a single 4-lobed or 4-angled bundle, and with 2-thecous anthers; usually multilocular ovaries and stigmas with distinct or weak lobes and smooth or rough; and fruits with a smooth surface. Three associated synonyms of *G.celebica*: *G.ferrea*, *G.basacensis*, and *G.hombroniana*, are lectotypified here in a second-step. In Thailand, *Garciniacelebica* is found in a very wide variety of habitats, at elevations of 0–1,500 m amsl., and is known to be naturally distributed in all floristic regions. *G.exigua* is found in dry evergreen forest on limestone hills and in littoral dry evergreen forest on limestone hills, at elevations of 50–100 m amsl. in Krabi Province, the peninsular region. G.mangostanavar.mangostana is found only in cultivation. *Garciniaexigua* has a conservation status of Vulnerable [VU B2ab(iii)] and the other two species have a conservation status of Least Concern [LC]. The fleshy pulp surrounding the seeds of two species, *G.celebica* and G.mangostanavar.mangostana is edible and has a sweet-sour taste.

## ﻿Introduction

*Garcinia* L. is the largest genus in the Clusiaceae Lindl. (Guttiferae Juss.). The genus contains at least 250 species (Stevens 2007) and maybe as many as c. 400 species (POWO 2024). It is a pantropically distributed genus and has centers of diversity located in Africa (Madagascar), Australasia, and Southeast Asia (Sweeney and Rogers 2008; Gaudeul et al. 2024). In Asia, *Garcinia* is most diverse in the Malesian region but also spreads north into southern China, west to India, and east to the Micronesian islands (Nazre et al. 2018). The genus is a group of evergreen small to large trees, or occasionally shrubs, which are usually dioecious, but sometimes polygamo-dioecious (also called trioecious). It also has obligately and facultatively agamospermous species (e.g., *G.mangostana* L.). Several species are well known because they have edible fruits or leaves (e.g., *G.atroviridis* Griff. ex T. Anderson, *G.cowa* Roxb. ex DC., *G.dulcis* (Roxb.) Kurz, *G.lanceifolia* Roxb., *G.mangostana*, *G.pedunculata* Roxb. ex Buch.-Ham., *G.schomburgkiana* Pierre) and used for medicinal purposes.

*Garcinia* honours Laurentius Garcin (1683–1752) who was a Dutch army doctor and naturalist in the Dutch Indies (Indonesia) in the years 1720–1729. During his voyage to the Maluku Islands (also called the Moluccas), Indonesia, he examined the fruit-bearing tree which the locals called ‘mangoustan’ (mangosteen) and gave a description of the fruiting female specimen (Garcin 1733). The species was named *Garciniamangostana* by Linnaeus (1753) and is the type species of the genus. The genus is characterized by a dioecious habit (sometimes apparently polygamo-dioecious); yellow, pale yellow, white, cream, or clear latex secreted from cut boles, twigs, leaves, and fruits; terminal buds concealed between the bases of the uppermost pair of petioles; decussate leaves with scattered black or brown gland dots, or interrupted wavy lines of differing lengths; male flowers with many to numerous stamens untied into a column in the center of the flower, or into a variously lobed or angled, or into 4 or 5 separate bundles; berry fruits and seeds usually with thick or thin fleshy pulp (Ngernsaengsaruay et al. 2022a).

Engler’s (1893) monograph of the genus *Garcinia* recognized 34 sections. Engler’s work was an elaboration of Pierre (1882, 1883), who established the first monograph of *Garcinia* and used mainly flower and inflorescence characters to classify the species into 37 sections. The other monograph of the genus *Garcinia* is that of Vesque (1893), who used floral morphology and leaf anatomy to classify the species into three subgenera and nine sections. A worldwide sectional treatment of *Garcinia* was presented by Jones (1980), in an unpublished Ph.D. thesis in which the genus was classified into 14 sections based mainly on floral morphology, especially male flowers and pollen morphology. Jones’s (1980) treatment recognized 46 species in the section Garcinia. This section was recently monographed by Nazre et al. (2018), who based on molecular and morphological data, recognized 13 species, two of which have three varieties each. Several species are excluded from GarciniasectionGarcinia, reported as insufficiently known, or reduced to synonymy (Nazre et al. 2018). The latest infrageneric classification of *Garcinia* was presented by Gaudeul et al. (2024), who recovered nine major clades falling within two major lineages, and recognized 11 sections, and recognized 15 species in section Garcinia. The section is distinguished by its flowers with 4 sepals and 4 petals; male flowers often with a pistillode, and stamens united into a single 4-lobed or 4-angled bundle, and with 2-thecous anthers; multilocular ovaries and stigmas with or without lobes and smooth or corrugated; fruits with a smooth surface; and terminal inflorescences and comprised of simple cymes (Nazre et al. 2018). Species of GarciniasectionGarcinia are typically understorey trees in tropical rain forests and are distributed in Southeast Asia from eastern India to Malesia (Nazre et al. 2018).

A taxonomic revision of the genus *Garcinia* in Thailand has recently been undertaken by the first author as part of the Flora of Thailand. Ngernsaengsaruay and Suddee (2016, 2022) described additional new species: *G.nuntasaenii* Ngerns. & Suddee from north-eastern and *G.santisukiana* Ngerns. & Suddee from eastern Thailand, respectively. Ngernsaengsaruay (2022) recognized three species in GarciniasectionBrindonia (Thouars) Choisy in Thailand: *G.atroviridis*, *G.lanceifolia*, and *G.pedunculata*. Ngernsaengsaruay et al. (2022a, 2023a) published additional new species records from peninsular Thailand: *G.dumosa* King and *G.exigua* Nazre, respectively. Ngernsaengsaruay et al. (2022b) published *Garciniasiripatanadilokii* Ngerns., Meeprom, Boonthasak, Chamch. & Sinbumr. as a new species from Peninsular Thailand. Finally, GarciniasectionXanthochymus (Roxb.) Pierre (Clusiaceae) was revised for Thailand with four native species: *G.dulcis* (Roxb.) Kurz, *G.nervosa* (Miq.) Miq., *G.prainiana* King, and *G.xanthochymus* Hook. f. ex T. Anderson (Ngernsaengsaruay et al. 2023b).

From these publications, the genus has a total of c. 30 accepted species in Thailand. However, identifications mostly rely on the literature, and this is the case for GarciniasectionGarcinia, which has never been revised for Thailand. Therefore, in this paper, we provide here an updated account for section Garcinia in Thailand in order to present a taxonomic treatment that includes lectotypifications, detailed morphological descriptions, illustrations, and an identification key to the species, together with notes on distributions, habitats and ecology, phenology, conservation assessments, etymology, vernacular names, uses, and specimens examined.

## ﻿Materials and methods

Specimens collected for the Flora of Thailand were examined by consulting taxonomic literature (e.g., Anderson 1874; Kurz 1874, 1877; Pierre 1882, 1883; King 1890; Vesque 1893; Pitard 1910; Gagnepain 1943; Corner 1952; Maheshwari 1964; Ridley 1922; Backer and Bakhuizen van den Brink 1963; Whitmore 1973; Singh 1993; Nazre 2010; Nazre et al. 2018), and by comparing with herbarium specimens housed in the following herbaria: AAU, BK, BKF, BM, C, CMUB, K, P, PSU, QBG, SING, and those included in the virtual herbarium databases of A (https://kiki.huh.harvard.edu/databases/specimen_index.html), AAU (https://www.aubot.dk/search_form.php), BR (http://www.botanicalcollections.be), CAL (https://ivh.bsi.gov.in/phanerogams), E (https://data.rbge.org.uk/search/herbarium/), G (http://www.ville-ge.ch/cjb/), K (including K-W) (http://www.kew.org/herbcat), KUN (Kunming Institute of Botany, Chinese Academy of Sciences, http://nsii.org.cn/2017/), L (including U) (https://bioportal.naturalis.nl/), MPU (https://explore.recolnat.org), P (https://science.mnhn.fr/institution/mnhn/collection/p/item/search/form), The Wallich Catalogue Online (https://wallich.rbge.org.uk/), US (https://collections.nmnh.si.edu/search/botany/), and W (https://www.nhm-wien.ac.at/en/research/botany). All herbaria acronyms follow Thiers (2024, continuously updated). All specimens cited have been seen by the authors unless stated otherwise. The taxonomic history of the species was compiled using the taxonomic literature and online databases (IPNI 2024; POWO 2024). The morphological characters, distributions, habitats and ecology, phenology, and uses were described from historic and newly collected herbarium specimens and the author’s observations during field work. The vernacular names were compiled from the specimens examined and the literature (e.g., Ridley 1922; Corner 1952; Maheshwari 1964; Whitmore 1973; Verheij and Coronel 1992; Pooma and Suddee 2014). Thailand floristic regions follow Flora of Thailand. Vol. 4(3.3) (The Forest Herbarium, Department of National Parks, Wildlife and Plant Conservation 2023). The assessment of conservation status was performed following the IUCN Red List Categories and Criteria (IUCN Standards and Petitions Committee 2022) for a preliminary assessment of the conservation category in combination with GeoCAT analysis (Bachman et al. 2011) and field information. The calculation of Extent of Occurrence (EOO) and Area of Occupancy (AOO) are based on GeoCAT (https://www.kew.org/science/our-science/projects/geocat-geospatial-conservation-assessment tool).

## ﻿Results and discussion

### ﻿Taxonomic treatment

#### ﻿ Garcinia L. section Garcinia

Taxon classificationPlantaeMalpighialesClusiaceae

L., Sp. Pl. 1: 443. 1753; S. W. Jones, Morphology and Major Taxonomy of Garcinia (Guttiferae), Ph.D. Thesis (unpublished): 284. 1980; Nazre et al., Phytotaxa 373(1): 14. 2018; M. Gaudeul et al., PhytoKeys 239: 93. 2024.

275EF791-5280-56AF-A421-167B3ABB7801

##### Type.

*Garciniamangostana* L., Sp. Pl. 1: 443. 1753.

##### Description.

***Habit*** evergreen trees, sometimes with buttresses near the base of the main stem of large trees; latex yellow (i.e., *G.exigua* and G.mangostanavar.mangostana) or white, turning yellow (i.e., *G.celebica*), sticky; branches decussate, horizontal or nearly horizontal; branchlets 4-ridged, glabrous. ***Terminal bud*** concealed between the bases of the uppermost pair of petioles. ***Leaves*** decussate, small (i.e., *G.exigua*) or big (i.e., *G.celebica* and G.mangostanavar.mangostana); lamina coriaceous or thickly coriaceous, glabrous; secondary veins curving towards the margin and connected in distinct loops and united into one (i.e., *G.celebica* and *G.exigua*) or two intramarginal veins (i.e., G.mangostanavar.mangostana), with interrupted long wavy lines (glandular wavy lines, also called exudate containing canals) of differing lengths, running across the secondary veins to the apex or the margin; petiole grooved or not grooved above, transversely rugose, usually with a basal appendage clasping the branchlets. ***Inflorescences*** terminal, simple cymes, in a cluster of two to several flowers, or sometimes a solitary flower (in the female flowers). ***Flowers*** unisexual, plants dioecious, 4-merous; bracteoles caducous; sepals and petals decussate. ***Male flowers***: stamens numerous, united into a single 4-lobed (i.e., *G.celebica* and *G.exigua*) or 4-angled bundle (i.e., G.mangostanavar.mangostana from Nazre et al. 2018), antepetalous (opposite the petals); anthers small, 2-thecous; pistillode present or absent. ***Female flowers***: staminodes absent or present; pistil fungiform (mushroom-shaped); ovary unlobed, usually multilocular; stigma sessile, distinctly or weakly lobed and smooth or rough. ***Fruits*** berries, subglobose, globose, depressed globose or broadly ellipsoid, small (i.e., *G.exigua*) or big (i.e., *G.celebica* and G.mangostanavar.mangostana), without or with a short beak at the apex, with thick or thin pericarp, turning woody when dry; persistent stigma flattened or slightly convex, distinctly or weakly lobed; persistent sepals usually larger than in flowering materials. ***Seeds*** (1–)4–9, usually with a fleshy pulp.

GarciniasectionGarcinia is characterized by its terminal inflorescences of simple cymes (in a cluster of two to several flowers), or sometimes a solitary flower (in the female flowers); flowers with 4 sepals and 4 petals; male flowers often with a pistillode, stamens united into a single 4-lobed or 4-angled bundle, and with 2-thecous anthers; usually multilocular ovaries, and stigmas with distinctly or weakly lobed and smooth or rough; and fruits with a smooth surface.

A section of 15 species worldwide (Gaudeul et al. 2024); three species in Thailand (i.e., two native species: *Garciniacelebica* L. and *G.exigua* Nazre, and one cultivated species and variety: G.mangostanaL.var.mangostana). Numbers of species in GarciniasectionGarcinia recognized by Jones (1980), Nazre et al. (2018), and Gaudeul et al. (2024) is shown in Table 1.

**Table 1.** Numbers of species in GarciniasectionGarcinia recognized by Jones (1980), Nazre et al. (2018), and Gaudeul et al. (2024).

Jones (1980)	Nazre et al. (2018)	Gaudeul et al. (2024)
–	1. *Garciniaacuticosta* Nazre	1. *Garciniaacuticosta* Nazre
1. *Garciniaaffinis* Wall.	*Garciniaaffinis* Wall. ex Pierre, *nom. illeg.* = *Garciniacelebica* L. (Nazre 2010)	–
2. *Garciniaanomala* Planch. & Triana	Excluded species	Unplaced species
3. *Garciniabaillonii* Pierre	–	–
4. *Garciniabasacensis* Pierre	= *Garciniacelebica* L.	–
5. *Garciniabenthamii* Pierre	= *Garciniacelebica* L.	–
6. *Garciniablancoi* Pierre	With unknown status	Unplaced species
7. *Garciniacalleryi* Pierre	–	–
8. *Garciniacelebica* L.	2. *Garciniacelebica* L.	2. *Garciniacelebica* L.
9. *Garciniachapelieri* (Planch. & Triana) H. Perrier	Excluded species	GarciniasectionBrindonia (Thoars) Choisy
10. *Garciniacornea* L.	= *Garciniacelebica* L.	–
11. *Garciniacostata* Hemsl. ex King	Excluded species	GarciniasectionBrindonia (Thoars) Choisy
12. *Garciniacumingiana* Pierre	–	–
13. *Garciniadiospyrifolia* Pierre	3a. *Garciniadiospyrifolia* Pierre var. *Diospyrifolia*	3. *Garciniadiospyrifolia* Pierre
–	3b. GarciniadiospyrifoliaPierrevar.cataractalis (Whitmore) Nazre	–
–	3c. GarciniadiospyrifoliaPierrevar.minor Ng ex Nazre	–
–	4. *Garciniadiscoidea* Nazre	4. *Garciniadiscoidea* Nazre
14. *Garciniaerythrosperma* Lauterb.	With unknown status	Unplaced species
–	5. *Garciniaexigua* Nazre	5. *Garciniaexigua* Nazre
15. *Garciniafabrilis* Miq.	= *Garciniacelebica* L.	–
16. *Garciniafascicularis* Wall.	–	–
17. *Garciniaferrea* Pierre	= *Garciniacelebica* L.	–
18. *Garciniaharmandii* Pierre	6. *Garciniaharmandii* Pierre	6. *Garciniaharmandii* Pierre
19. *Garciniahombroniana* Pierre	= *Garciniacelebica* L.	–
20. *Garciniajawoera* Pierre	= *Garciniacelebica* L.	–
21. *Garciniakingii* Pierre ex Vesque	= *Garciniacelebica* L.	–
22. *Garciniakrawang* Pierre	= *Garciniacelebica* L.	–
23. *Garciniakurzii* Pierre	= *Garciniacelebica* L.	–
24. *Garcinialucens* Pierre	With unknown status	Unplaced species
25. *Garciniamacrophylla* (Miq.) Miq.	–	–
26. *Garciniamaingayi* Hook. f.	Excluded species	GarciniasectionBrindonia (Thoars) Choisy
27. *Garciniamalaccensis* Hook. f.	= *Garciniamangostana* L. *malaccensis* (Hook. f.) Nazre	–
28. *Garciniamangostana* L.	7a. *Garciniamangostana* L. var. *Mangostana*	7. *Garciniamangostana* L.
–	7b. GarciniamangostanaL.var.malaccensis (Hook. f.) Nazre	–
–	7c. GarciniamangostanaL.var.borneensis Nazre	–
–	–	8. *Garciniamangostifera* Kaneh. & Hatus.
29. *Garciniamoselleyana* Pierre	Excluded species	GarciniasectionMacrostigma Pierre
30. *Garciniamoulmeinensis* Pierre ex Vesque	With unknown status	Unplaced species
31. *Garcinianitida* Pierre	8. *Garcinianitida* Pierre	9. *Garcinianitida* Pierre
–	9. *Garciniaochracea* Nazre	10. *Garciniaochracea* Nazre
32. *Garciniaopaca* King	= *Garciniadiospyrifolia* Pierre	–
33. *Garciniapenangiana* Pierre	10. *Garciniapenangiana* Pierre	11. *Garciniapenangiana* Pierre
34. *Garciniaporrecta* Wall.	*Garciniaporrecta* Wall. ex Vesque = *Garciniacelebica* L. (Nazre 2010)	–
35. *Garciniapropinqua* Craib	Excluded species	Unplaced species
36. *Garciniapseudoguttifera* Seem.	Excluded species	–
37. *Garciniariedeliana* Pierre	= *Garciniacelebica* L.	–
38. *Garciniarigida* Miq.	11. *Garciniarigida* Miq.	12. *Garciniarigida* Miq.
39. *Garciniarumphii* Pierre	= *Garciniacelebica* L.	–
–	12. *Garciniasangudsangud* Nazre	13. *Garciniasangudsangud* Nazre
40. *Garciniaschefferi* Pierre	–	–
–	–	14. *Garciniasibeswarii* Shameer, J. Sarma, N. Mohanan & A. Begum
41. *Garciniaspeciosa* Wall.	= *Garciniacelebica* L.	–
42. *Garciniasquamata* Lauterb.	With unknown status	Unplaced species
43. *Garciniatonkinensis* Vesque	Excluded species	–
44. *Garciniatrianii* Pierre	Excluded species	–
45. *Garciniavenulosa* (Blanco) Choisy	13. *Garciniavenulosa* (Blanco) Choisy	15. *Garciniavenulosa* (Blanco) Choisy
46. *Garciniavidua* Ridl.	Excluded species	–
46 species	13 species, two of which have three varieties	15 species

### ﻿A key to the species of GarciniasectionGarcinia in Thailand

**Table:** 

1	Leaves more than 6.3 × 3.2 cm, tough when crushed (in fresh leaves); petiole more than 10 × 1.5 mm; fruits more than 1.3 × 1.1 cm; bark scaly or fissured	**2**
–	Leaves up to 6.3 × 3.2 cm, brittle when crushed (in fresh leaves); petiole up to 10 × 1.5 mm in diam.; fruits up to 1.3 × 1.1 cm; bark mottled, flaking and leaving roundish or irregularly shaped scars	**2. *Garciniaexigua***
2	Leaves with one intramarginal vein; petiole 2–4 mm in diam., grooved; mature flower buds up to 1 cm in diam.; female flowers 2–2.8 cm in diam.; petals creamish white or pale yellow; stigma shallowly lobed (also seen in fruiting materials); fruits yellow, orange, reddish orange to red when ripe, broadly ellipsoid, subglobose, globose or depressed globose, without or with a short, thick beak at the apex; persistent sepals usually up to 1.5 × 1.4 cm (in fruiting materials); latex white, turning yellow; found in the wild	**1. *Garciniacelebica***
–	Leaves with two intramarginal veins; petiole 4–7 mm in diam., not grooved; mature flower buds more than 1 cm in diam.; female flowers 3.2–5 cm in diam.; petals yellowish red or yellowish pink; stigma deeply lobed (also seen in fruiting materials); fruits pinkish pale yellow, pink, reddish purple to blackish purple when ripe, subglobose or globose, without a beak at the apex; persistent sepals usually up to 2.5 × 2.8 cm (in fruiting materials); latex yellow; found only in cultivation	**3. Garciniamangostanavar.mangostana**

#### ﻿1. Garcinia celebica

Taxon classificationPlantaeMalpighialesClusiaceae

L., Herb. Amboin.: 7. 1754; DC., Prodr. 1: 561. 1824; Miq. Fl. Ned. Ind. 1(2): 507. 1859; Planch. & Triana, Ann. Sci. Nat., Bot., sér. 4, 14: 328. 1860; Pierre, Fl. Forest. Cochinch. 1(5): 13. 1883; Vesque in A. DC. & C. DC., Monogr. Phan. 8: 404. 1893; Engl. in Engl. & Prantl, Die Naturlichen Pflanzenfamilien 3(6): 236. 1893; Koord. & Valeton, Bijdr. Boomsoort. Java 9: 367. 1903; Backer & Bakh. f., Fl. Java (Spermatoph.) 1: 387. 1963; Nazre, Genet. Resour. Crop. Evol. 57: 1256. 2010; Nazre et al., Phytotaxa 373(1): 17. 2018.

4C5CC22B-E929-51CF-A92E-5818863A47A5

Figs 1 , 2 , 3

≡ Brindoniacelebica (L.) Thouars in F. Cuvier, Dict. Sci. Nat. 5: 341. 1806.
≡ Oxycarpuscelebica (L.) Poir., Encyc. Suppl. 4: 258. 1816.
≡ Stalagmitiscelebica (L.) G. Don, Gen. Hist. 1: 621. 1831. Type. Rumphius’s illustration, Mangostanacelebica Rumph., Herb. Amboin. 1: 134. t. 44 (Rumphius 1741) (lectotype, designated by Merrill 1917: 373).
= Garciniacornea L., Syst. Veg., ed. 13. 368. 1774; Blume, Bijdr. Fl. Ned. Ind.: 214. 1825; G. Don, Gen. Hist. 1: 620. 1831; Roxb. in Carey, Fl. Ind. 2: 629. 1832; Wight, Icon. Pl. Ind. Orient. 1(10): 6. t. 105. 1839; Miq., Fl. Ned. Ind. 1(2): 506. 1859; Planch. & Triana, Ann. Sci. Nat., Bot., sér. 4, 14: 325. 1860; Laness., Mém. Gen. Garc.: 20. 1872; Kurz, J. Asiat. Soc. Bengal, Pt. 2, Nat. Hist. 43(2): 86. 1874 et Forest Fl. Burma 1: 88. 1877; Pierre, Fl. Forest. Cochinch. 1(5): 12. t. 78B. 1883; Vesque in A. DC. & C. DC., Monogr. Phan. 8: 397. 1893; Engl. in Engl. & Prantl, Die Naturlichen Pflanzenfamilien 3(6): 236. 1893; Merr., Interpr. Herb. Amboin.: 374. 1917; Maheshw., Bull. Bot. Surv. India 6: 122. t. 2. fig. 16. 1964; Nazre, Genet. Resour. Crop. Evol. 57: 1256. 2010. Type. Rumphius’s illustration, Lignumcorneum Rumph., Herb. Amboin. 3: 55. t. 30 (Rumphius 1743) (lectotype, designated by Merrill 1917: 374).
= Garciniaaffinis Wall. [Numer. List: 171. Wallich Cat. 4854. 1831, *nom. nud.*] ex Pierre, Fl. Forest. Cochinch. 1(5): 16. t. 78C, 79G. 1883, *nom. illeg.*
= Garciniaspeciosa Wall., Pl. Asiat. Rar. 3: 37. 1832; Planch. & Triana, Ann. Sci. Nat., Bot., sér. 4, 14: 326. 1860; T. Anderson in Hook. f., Fl. Brit. India 1(2): 260. 1874; Kurz, J. Asiat. Soc. Bengal, Pt. 2, Nat. Hist. 43(2): 86. 1874 et Forest Fl. Burma 1: 88. 1877; Pierre, Fl. Forest. Cochinch. 1(5): 14. t. 79H, I. 1883; King, J. Asiat. Soc. Bengal, Pt. 2, Nat. Hist. 59(2): 154. 1890; Vesque in A. DC. & C. DC., Monogr. Phan. 8: 402. 1893; Engl. in Engl. & Prantl, Die Naturlichen Pflanzenfamilien 3(6): 236. 1893; Brandis, Indian Trees: 50. 1906; C. E. Parkinson, Forest Fl. Andaman Isl.: 90. 1923; Craib, Fl. Siam. 1(1): 117. 1925; Gagnep. in Gagnep., Fl. Indo-Chine Suppl.: 267. 1943; Maheshw., Bull. Bot. Surv. India 6: 123. t. 2. fig. 18. 1964; N. P. Singh in B. D. Sharma & Sanjappa, Fl. Ind. 3: 125. 1993; S. Gardner, P. Sidisunthorn & V. Anusarnsunthorn, Field Guide Forest Trees of N. Thailand: 50. fig. 53. 2000; Nazre, Genet. Resour. Crop. Evol. 57: 1256. 2010; S. Gardner, P. Sidisunthorn & Chayam., Forest Trees S. Thailand 1: 355. fig. 545. 2015. Type. Myanmar, Amherst, 1827, *Wallich Cat. 4855* (lectotype, designated by Maheshwari 1964: 123), CAL [CAL0000065160, photo seen]; isolectotype K-W [K001104074!]).
= Garciniafabrilis Miq., Fl. Ned. Ind., Eerste Bijv. 3: 496. 1861 [as Discostigmafebrile]; Pierre, Fl. Forest. Cochinch. 1(5): 15. t. 80A. 1883; Vesque in A. DC. & C. DC., Monogr. Phan. 8: 401. 1893. Type. Indonesia, Sumatra, Priaman, s.d., *Diepenhorst HB2152* (lectotype, designated by Nazre 2010: 1256), L [U1572338, photo seen]).
= Garciniabenthamii Pierre, Fl. Forest. Cochinch. 1(4): t. 55, 56. 1882 [as Garciniabenthami]; Vesque, Epharmosis 2: 18. t. 109, 110. 1889 et in A. DC. & C. DC., Monogr. Phan. 8: 392. 1893 [as G.benthami]; Merr., Philipp. J. Sci. 3: 364. 1908 [as G.benthami]; Pit. in Lecomte et al., Fl. Indo-Chine 1(4): 305. 1910 [as G.benthami]; Merr., Enum. Philipp. Fl. Pl. 3: 83. 1923 [as G.benthami]; Gagnep. in Gagnep., Fl. Indo-Chine Suppl.: 261. 1943 [as G.benthami]; P. H. Hô, Câyco Vietnam 1: 561. fig. 1550. 1991 [as G.benthami]. Type. Vietnam, ad Bung in prov. Saïgon, Jan 1875, *Pierre 700* (lectotype, designated by Nazre 2010: 1256), P [P00329872, photo seen]; isolectotypes A [without barcode, reported by Nazre 2010 and Nazre et al. 2018, not seen], K [without barcode, reported by Nazre 2010 and Nazre et al. 2018, not seen], L [U1208099, U1208248, photos seen], P [P04701491, photo seen].
= Garciniaferrea Pierre, Fl. Forest. Cochinch. 1(4): t. 57. 1882; Vesque, Epharmosis 2: 18. t. 110, 111. 1889; Engl. in Engl. & Prantl, Die Naturlichen Pflanzenfamilien 3(6): 236. 1893; Pit. in Lecomte et al., Fl. Indo-Chine 1(4): 303. 1910; Gagnep. in Gagnep., Fl. Indo-Chine Suppl.: 261. 1943; Pételot, Arch. Rech. Agron. Cambodge Laos Vietnam 1: 60. 1952; P. H. Hô, Câyco Vietnam 1: 562. fig. 1553. 1991. Type. Vietnam, Phu Quoc, Jan 1877, *Herb. Pierre 3634* (lectotype, first-step designated by Nazre 2010: 1256), P [without barcode], second-step designated here P [P00379823!]; isolectotype P [P00379824!]).
= Garciniabasacensis Pierre, Fl. Forest. Cochinch. 1(4): t. 58. 1882 [as G.bassacensis]; Vesque in A. DC. & C. DC., Monogr. Phan. 8: 398. 1893 [as G.bassacensis]; Pit. in Lecomte et al., Fl. Indo-Chine 1(4): 306. 1910 [as G.bassacensis]; P. H. Hô, Câyco Vietnam 1: 561. fig. 1549. 1991 [as G.bassacensis]. Type. Laos, Bassin d’Attopeu, Mont de Bassac, Feb 1877, *Harmand 1074* (lectotype, first-step designated by Nazre 2010: 1256), P [without barcode], second-step designated here P [P00329871!]; isolectotypes K [K000380454!], P [P00329870!, P05062473!]).
= Garciniariedeliana Pierre, Fl. Forest. Cochinch. 1(5): 12. t. 79A. 1883; Vesque, Epharmosis 2: 18. t. 156. 1889 et in A. DC. & C. DC., Monogr. Phan. 8: 388. 1893. Type. Indonesia, Sulawesi, Gorontalo, 1875, *Riedel s.n.* (lectotype, designated by Nazre et al. 2018: 17), K [K000380456, photo seen]; isolectotypes P [P04700635!, P04700640!, P04700639, photo seen]).
= Garciniahombroniana Pierre, Fl. Forest. Cochinch. 1(5): 12. t. 79D–F, J. 1883; Vesque, Epharmosis 2: 18. t. 113. 1889; King, J. Asiat. Soc. Bengal, Pt. 2, Nat. Hist. 59(2): 155. 1890; Vesque in A. DC. & C. DC., Monogr. Phan. 8: 395. 1893; Engl. in Engl. & Prantl, Die Naturlichen Pflanzenfamilien 3(6): 236. 1893; Ridl., Fl. Malay Penins. 1: 171. 1922; Craib, Fl. Siam. 1(1): 115. 1925; Corner, Wayside Trees Mal. 1: 318. fig. 109. ed. 2. 1952; Maheshw., Bull. Bot. Surv. India 6: 121. t. 2. fig. 15. 1964; Corner & Watan., Ill. Guide Trop. Pl.: t. 190. 1969; Whitmore in Whitmore, Tree Fl. Malaya 2: 212. fig. 7. 1973; S. W. Jones, Morphology and Major Taxonomy of Garcinia (Guttiferae), Ph.D. Thesis (unpublished): 290. 1980; H. Keng, Concise Fl. Singapore: 49. 1990; N. P. Singh in B. D. Sharma & Sanjappa, Fl. Ind. 3: 111. 1993; M. Turner, Gard. Bull. Singapore 47(1): 262. 1995; S. Gardner, P. Sidisunthorn & Chayam., Forest Trees S. Thailand 1: 354. fig. 544. 2015. Type. Peninsular Malaysia, Malacca, 1841, *J. B. Hombron s.n.* (lectotype, first-step designated by Nazre 2010: 1256), P [without barcode], second-step designated here P [P00329889!]; isolectotypes P [P00329878, P04700177, P04700178, P04700180, P04700181, P04700182, photos seen]).
= Garciniarumphii Pierre, Fl. Forest. Cochinch. 1(5): 13. t. 77A. 1883; Vesque, Epharmosis 2: 18. t. 114. 1889 et in A. DC. & C. DC., Monogr. Phan. 8: 400. 1893. Type. Indonesia, Bangka Island, Nov 1881, *Treub 4169* (lectotype, first-step designated by Nazre 2010: 1256–1257), P [without barcode], second-step designated by Nazre et al. 2018: 17, P [P04700302!]; isolectotypes K [K000380451, photo seen], P [P04700298!, P04700299!]).
= Garciniakurzii Pierre, Fl. Forest. Cochinch. 1(5): 14. t. 78A. 1883; Vesque, Epharmosis 2: 18. t. 114. 1889; King, J. Asiat. Soc. Bengal, Pt. 2, Nat. Hist. 59(2): 155. 1890; Vesque in A. DC. & C. DC., Monogr. Phan. 8: 403. 1893; Maheshw., Bull. Bot. Surv. India 6: 123. 1964; N. P. Singh in B. D. Sharma & Sanjappa, Fl. Ind. 3: 115. 1993. Type. India, South Andaman, 1867, *Kurz 24* (lectotype, designated by Maheshwari 1964: 123), CAL [without barcode, not seen]; isolectotypes P [P00329891!, P00329890, photos seen]).
= Garciniajawoera Pierre, Fl. Forest. Cochinch. 1(5): 37. 1883; Vesque in A. DC. & C. DC., Monogr. Phan. 8: 399. 1893. Type. Indonesia, Java, Tandjoor, cultivated in Hort. Bog., 1877, *Pierre 4607* (lectotype, first-step designated by Nazre 2010: 1257), P [without barcode], second-step designated by Nazre et al. 2018: 17), P [P00379817!]; isolectotypes L [without barcode, reported by Nazre 2010 and Nazre et al. 2018, not seen], P [P00379816!, P00379818!]).
= Garciniakrawang Pierre, Fl. Forest. Cochinch. 1(5): 37. 1883; Vesque in A. DC. & C. DC., Monogr. Phan. 8: 398. 1893. Type: Indonesia, Borneo, South Kalimantan, Pulau Lampei (Lampei Island), s.d., *Korthals 1313a* (*Herb. Pierre 4601*) (lectotype, first-step designated by Nazre 2010: 1257), P [without barcode], second-step designated by Nazre et al. 2018: 17), P [P00379812, photo seen]; isolectotype P [P00379813, photos seen]).
= Garciniakingii Pierre ex Vesque in A. DC. & C. DC., Monogr. Phan. 8: 407. 1893; Maheshw., Bull. Bot. Surv. India 6: 124. 1964; N. P. Singh in B. D. Sharma & Sanjappa, Fl. Ind. 3: 114. 1993. Type: Andaman Island, 1884, *King’s Collector s.n.* (lectotype, designated by Nazre 2010: 1257), K [K000380453, photo seen]).

##### Description.

***Habit*** trees, 5–25-(–30) m tall, 30–150(–200) cm GBH, sometimes with buttresses near the base of the main stem of large trees; latex white, turning yellow, sticky; branchlets green, 4-ridged, glabrous. ***Bark*** greyish brown, brown, dark brown or blackish brown, scaly or fissured; inner bark reddish pink or red. ***Leaves***: lamina variable in shape and size, elliptic, oblong-elliptic, ovate, elliptic-ovate or lanceolate-ovate, 10–24 × 4–9.5 cm, apex acute, base cuneate or oblique, margin repand or undulate, thickly coriaceous, smooth, shiny dark green above, paler below, glabrous on both surfaces, midrib raised on both surfaces, secondary veins 12–25 each side, curving towards the margin and connected in distinct loops and united into an intramarginal vein, flattened on both surfaces, intramarginal veins not grooved above, with intersecondary veins, veinlets reticulate, visible below, interrupted long wavy lines of differing lengths, running across the secondary veins to the apex, conspicuous below; petiole green, stout, 1–2 cm long, 2–4 mm in diam., grooved above, distinctly transversely rugose, glabrous, with a basal appendage clasping the branchlet; young leaves brownish green, turning pale green, glossy; fresh leaves tough when crushed; mature leaves turning greenish yellow to pale yellow before falling off; dry leaves pale brown or reddish brown. ***Inflorescences*** terminal; bracts 2, caducous, narrowly triangular or triangular, 0.8–1.3 × 0.1–0.4 cm, apex acute (in female inflorescences). ***Flowers***: sepals and petals glabrous; sepals concave; petals creamish white or pale yellow, somewhat fleshy, concave or not concave, apex rounded, margin entire or irregularly lobed and undulated. ***Flower buds*** subglobose to globose, 0.5–1 cm in diam. ***Male flowers*** lightly fragrant, in a cluster of 2–7 flowers, 1.8–2.5 cm in diam.; bracteoles caducous; pedicel pale yellow, reddish pale yellow or yellowish red, slender, terete (circular in cross-section) or slightly 4-angled, 0.4–1 cm long, 1.5–3 mm in diam., glabrous; sepals 4, pale yellow, reddish pale yellow or yellowish red, thinly coriaceous, broadly elliptic, elliptic, suborbicular or orbicular, 0.5–1 × 0.4–1 cm, the outer pair slightly smaller than the inner pair, apex rounded; petals 4, suborbicular, orbicular, broadly elliptic or elliptic, 0.7–1.2 × 0.5–1.1 cm, subequal; stamens 144–198, united into a single 4-lobed bundle (35–53 each lobe), surrounding a pistillode, lobes 5–9 × 5–8.5 mm; filaments very short; anthers 1–2 × 0.5–1 mm; pistillode fungiform, 5–7.5 mm long; sterile stigma pale yellow or yellow, sessile, convex, radiate, shallowly 4–9-lobed, 3.5–5 mm in diam., smooth. ***Female flowers*** solitary or in a cluster of 2–3 flowers, 2–2.8 cm in diam.; bracteoles caducous, triangular, 1.5–5 × 1–4 mm; pedicel (of a flower in an inflorescence) or peduncle (of a solitary flower) green, stout, terete or slightly 4-angled, 0.4–1.5 cm long, 2–4 mm in diam., glabrous; sepals 4, pale green, thickly coriaceous, suborbicular, orbicular or broadly elliptic, 0.4–1.2 × 0.4–1.2 cm, the outer pair slightly smaller than the inner pair, apex rounded; petals 4, suborbicular, orbicular, broadly elliptic or elliptic, 0.7–1.5 × 0.7–1.3 cm, subequal; staminodes absent or present, united into 9–15 bundles, surrounding the ovary, each bundle 1–2 mm long; pistil fungiform, 0.5–1 cm long; ovary pale green, subglobose or globose, 4–6.5 × 4–7.5 mm, glabrous, 4–9-locular; stigma pale yellow or yellow, convex, radiate, shallowly 4–9-lobed, 2–4 mm long, 5.5–8 mm in diam., smooth. ***Fruits*** pale green, turning yellow, orange, reddish orange to red when ripe, smooth, glabrous, with a sticky white latex, turning yellow, broadly ellipsoid, subglobose, globose or depressed globose, 1.8–5.5 × 2–5.6 cm (length including a beak), without or with a short, thick beak at the apex, 2.5–6.5 × 5–11.5 mm, pericarp 2–7 mm thick, fleshy, becoming woody when dry; persistent stigma dark brown or blackish brown, flattened, radiate, shallowly 4–9-lobed, 0.4–1.1 cm in diam.; persistent sepals green or green tinged with red, turning yellowish green to yellow or yellow tinged with red, thickly coriaceous, 0.5–1.5 × 0.5–1.4 cm, usually larger than in flowering materials; fruiting stalk green, strong and thick, 0.5–1.7 cm long, 2.5–6.5 mm in diam., glabrous. ***Seeds*** 4–9, sometimes aborted, brown mottled with irregular lines, ellipsoid or broadly ellipsoid, 0.7–2.4 × 0.4–1.6 cm, compressed, rounded at both ends, with a white fleshy pulp.

**Figure 1.**
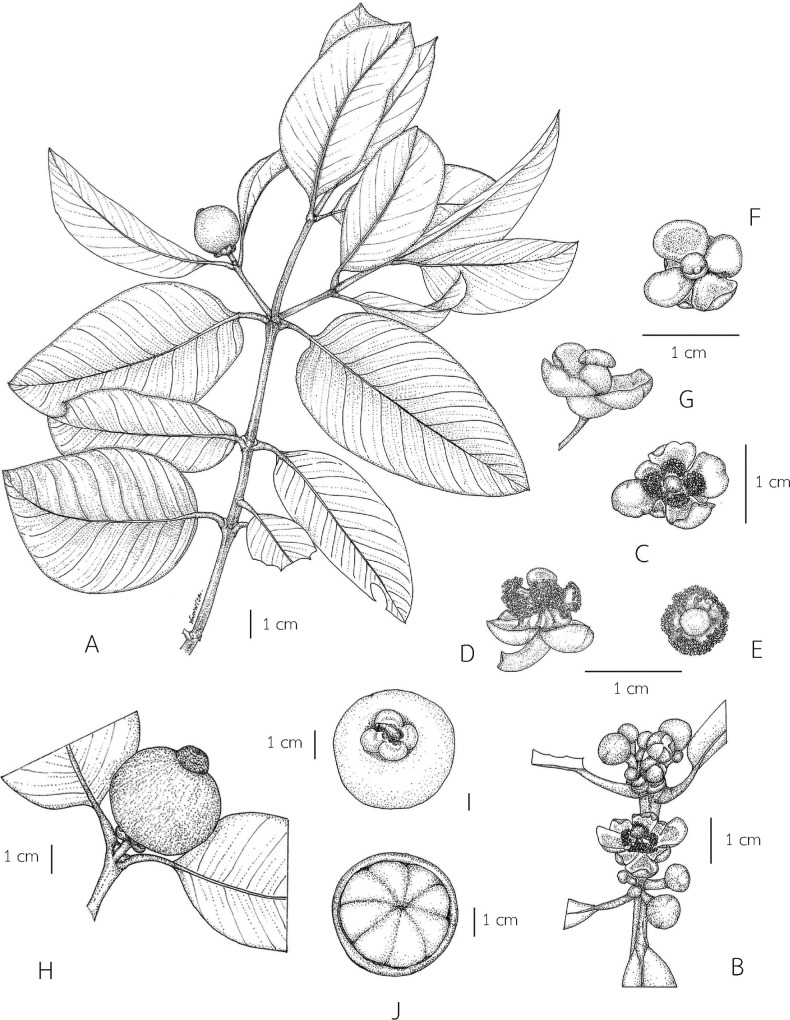
*Garciniacelebica***A** branchlets, leaves, and fruit **B** branchlets and male inflorescences with male flower buds and male flower **C** male flower (top view) **D** male flower (side view) **E** male flower showing 4-lobed stamen bundle and a pistillode (sepals and petals removed) **F** female flower (top view) **G** female flower (side view) **H** branchlet and fruit **I** fruit showing persistent sepals **J** fruit (transverse section) and seeds with a fleshy pulp. Photo: Drawn by Wanwisa Bhuchaisri.

##### Distribution.

India [North-Eastern India (Assam, Meghalaya, West Bengal), Andaman and Nicobar Islands], Bangladesh, Myanmar (Martaban, Tenasserim), Vietnam, Laos, Cambodia, Thailand, Peninsular Malaysia (Perlis, Kedah, Penang, Perak, Terengganu, Pahang, Selangor, Malacca), Singapore, Indonesia (Sumatra, Java, Lesser Sunda Islands, Sulawesi, Maluku), Borneo [Malaysia (Sarawak, Sabah), Brunei, Indonesia (Kalimantan)], Philippines (Luzon, Palawan, Mindanao), New Guinea [Indonesia (Western New Guinea), Papua New Guinea].

**Figure 2.**
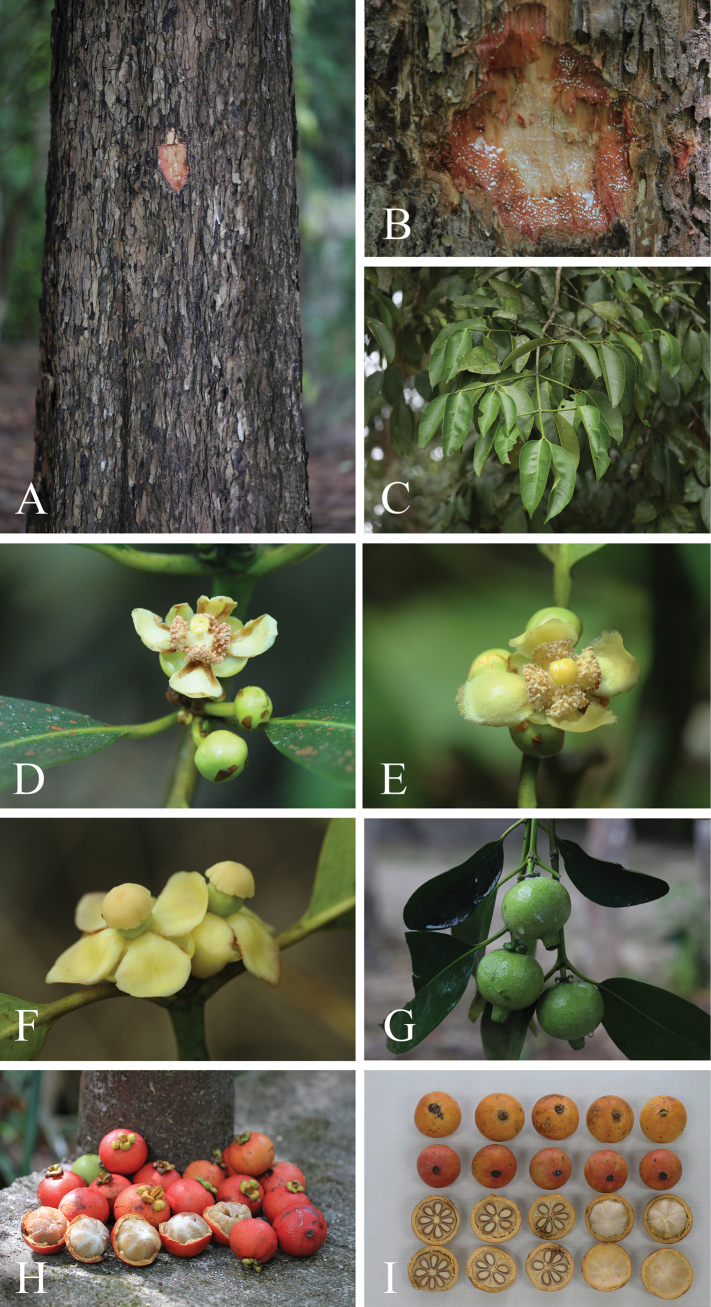
*Garciniacelebica***A** stem and bark **B** slashed bark with white latex **C** branchlets and leaves **D, E** branchlets and male inflorescences with male flower buds and male flowers **F** branchlet and female inflorescence with female flowers **G** branchlets, leaves, and mature fruits **H, I** mature and ripe fruits and seeds with a white fleshy pulp. Photos: Chatchai Ngernsaengsaruay.

##### Distribution in Thailand.

**Northern**: Chiang Mai, Chiang Rai, Phayao, Phrae, Phitsanulok; **North-Eastern**: Loei, Nong Khai, Bueng Kan, Sakon Nakhon, Khon Kaen; **Eastern**: Chaiyaphum, Ubon Ratchathani; **South-Western**: Uthai Thani, Kanchanaburi, Phetchaburi, Prachuap Khiri Khan; **Central**: Saraburi; **South-Eastern**: Sa Kaeo, Prachin Buri, Chon Buri, Chanthaburi, Trat; **Peninsular**: Chumphon, Ranong, Surat Thani, Phangnga, Krabi, Nakhon Si Thammarat, Trang, Satun, Songkhla, Pattani, Yala, Narathiwat.

**Figure 3.**
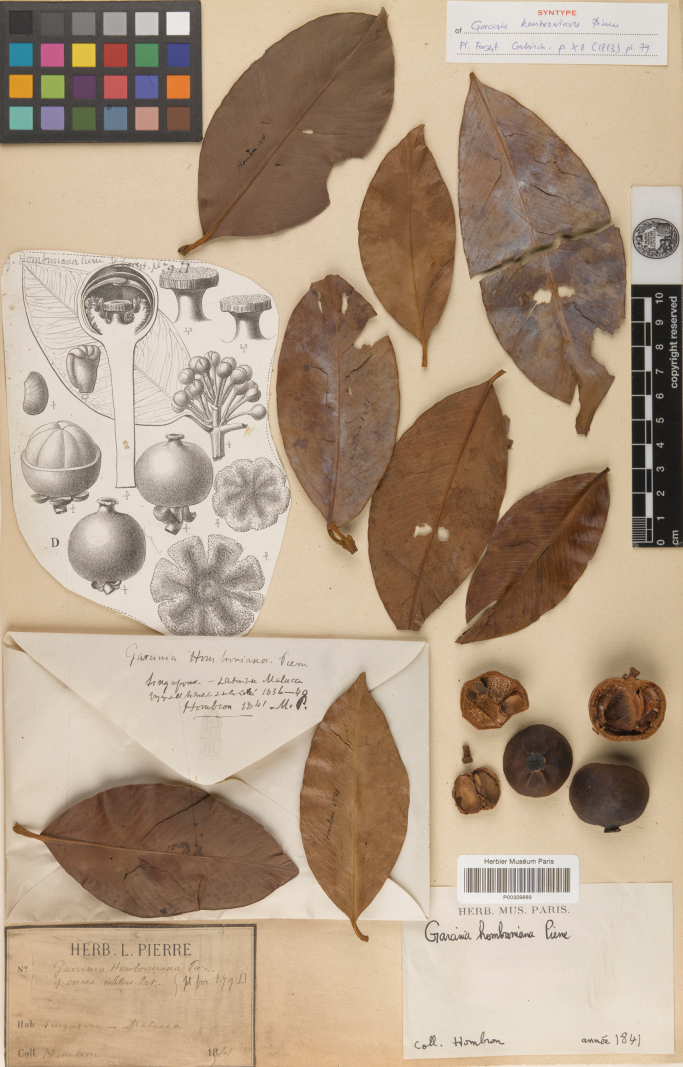
Lectotype of *Garciniahombroniana*, a synonym of *Garciniacelebica*, *J. B. Hombron s.n.* (P [P00329889]) from Malacca, Peninsular Malaysia, second-step lectotype designated here. Photo: Muséum National d’Histoire Naturelle, Paris, France, http://coldb.mnhn.fr/catalognumber/mnhn/p/p00329889.

##### Habitat and ecology.

This species is found in a very wide variety of habitats, including coastal strand vegetations, littoral dry evergreen forests, dry evergreen forests, tropical evergreen rain forests, freshwater swamp forests, lower montane rain forests, lower montane coniferous forests, pine-deciduous dipterocarp forests, mixed deciduous forests, secondary forests, on limestones, on sandstone plateaus, sometimes along streams, 0–1,500 m amsl.

##### Phenology.

Flowering and fruiting more than once, nearly throughout the year; flowering usually in November to February; fruiting usually in February to May.

##### Conservation status.

*Garciniacelebica* is widely distributed from Eastern India to the Malesian region. It is known from many localities and has a large Extent of Occurrence (EOO) of 21,968,911.92 km^2^ and a relatively large Area of Occupancy (AOO) of 700 km^2^. In Thailand, this species is known to be naturally distributed throughout the seven floristic regions, and has an EOO of 427,003.87 km^2^ and an AOO of 96 km^2^. Because of this wide distribution and the number of localities, therefore, we consider the conservation assessment here as Least Concern (LC).

##### Etymology.

The specific epithet of *Garciniacelebica* indicates the type locality, Sulawesi (formerly known as Celebes), Indonesia (Nazre 2010), as described by Rumphius (1741). The specific epithet of *G.speciosa* is a Latin word meaning showy or splendid (Stearn 1992; Gledhill, 2002) and refers to the ripe fruits are orangish red to red. The specific epithet of *G.hombroniana* is named after J. B. Hombron, a French physician and explorer who collected the type specimen during his journey from Singapore to Malacca (Peninsular Malaysia) (Corner 1952; Whitmore 1973; Nazre 2010).

##### Vernacular names.

Kwak mai (กวกไหม) (Bueng Kan, Nongkhai, Laos); Kawa (กะวา) (Surat Thani); Khwat (ขวาด) (Chiang Rai, Laos); Chamuang (ชะม่วง) (Phichit); **Phawa** (พะวา) (Surat Thani); Mada khinok (มะดะขี้นก) (Chiang Mai, Laos); Mapong (มะป่อง) (Northern); Mangkhut pa (มังคุดป่า) (Narathiwat); Wa (วา) (Phangnga, Songkhla, Surat Thani, Yala); Wa nam (วาน้ำ) (Trang); Sommong Pa (ส้มโมงป่า) (Nongkhai); Saraphi pa (สารภีป่า) (Central, Chiang Mai); Mak kwak (หมากกวก) (Bueng Kan, Nongkhai, Laos); Beruas, Bruas, Mangis hutan (Peninsular Malaysia); Parawa (Myanmar); Jungle mangosteen, Seashore mangosteen (English).

##### Uses.

The fleshy pulp surrounding the seeds can be consumed and has a sweet-sour taste. The wood is used for house construction, making oars (Maheshwari 1964; Burkill et al. 1966), the handles of the tools, and bridge posts (Maheshwari 1964). In Andaman Islands, the wood is used for making bows (Maheshwari 1964). In Malaysia, *Garciniacelebica* have been recorded at more than 50 cm dbh and may be logged for timber (Nazre et al. 2018). The fruits are reported to cause constipation (Sastri 1956). In Peninsular Malaysia, the roots and leaves are used to relieve itching (Sastri 1956; Maheshwari 1964). A decoction of the root may be administered after childbirth as a preventive medicine (Burkill et al. 1966).

##### Lectotypifications.

*Garciniaferrea* was named by Pierre (1882: t. 57), who cited three gatherings: *Herb. Pierre 3634*, *3635*, and *3695* but he did not mention the name of the herbarium where the materials were kept, and following Art. 9.6 of the ICN (Turland et al. 2018), they constitute syntypes. The name *G.ferrea* has been lectotypified twice, first by Nazre (2010: 1256), who selected a specimen *Herb. Pierre 3634* in Cambodia, and deposited at P [without barcode], while Nazre et al. (2018: 17) chose a Cambodian specimen of Pierre (*Herb. Pierre 3635*) housed at P [P00329882]. Hence, the first lectotypification has priority (as the first-step). We located two sheets of the specimen *Herb. Pierre 3634* at P [P00379823, P00379824]; therefore, the P [P00379823] specimen is selected here in a second-step lectotypification (following Art. 9.17 and Ex. 14 of the Shenzhen Code). Incidentally, from our examination of specimens at P Herbarium, *Herb. Pierre 3634* (Phu Quoc) and *Herb. Pierre 3635* (in montibus Dinh ad Baria Gallicae Austro-Cochinchinae) were not collected from Cambodia as mentioned by Nazre (2010) and Nazre et al. (2018), but were collected in Vietnam.

In the original publication of *Garciniabasacensis* by Pierre (1882: t. 58), only one gathering is mentioned, *Hermand 1074* (*Herb. Pierre 3637*). The name *G.basacensis* has been lectotypified twice, firstly, Nazre (2010: 1256) lectotypified this name using the material at P [without barcode], with an isolectotype at K [without barcode] collected from Laos, and secondly, Nazre et al. (2018: 17) lectotypified this name using the same material. Therefore, the first lectotypification has priority (as the first-step). However, there are three sheets of this gathering at P [P00329870, P00329871, P05062473] and the P [P00329871] material is selected here in a second-step lectotypification. We located one sheet of isolectotype at K [K000380454].

*Garciniahombroniana* was named by Pierre (1883: 12. t. 79D–F, J) based on the specimens collected from Ile de Singapoor-Détroit de Malacca. He did not designate a holotype nor did he mention the name of the herbarium where the specimens were housed. The name *G.hombroniana* has been lectotypified twice, first by Nazre (2010: 1256), who designated the specimen collected by J. B. Hombron (*Hombron s.n.*) from Malacca housed in P [without barcode], and second by Nazre et al. (2018: 17), who selected the same specimen. Therefore, the first lectotypification has priority (as the first-step). We traced seven specimens of *J. B. Hombron s.n.* at P [P00329878, P00329889, P04700177, P04700178, P04700180, P04700181, P04700182]. The P [P00329889] specimen is better preserved and more complete than the others, and hence is chosen here as the second-step lectotype.

##### Notes.

According to Nazre et al. (2018), the shape and size of leaves of *Garciniacelebica* are elliptic, broadly elliptic, lanceolate, sub-orbiculate or round and 3.3–17.5 × 1.7–11 cm; the male inflorescences in clusters of 2–14(–18) flowers; the staminodes are absent; the ovaries have 4–6 locules; and the shape and size of fruits are ovoid, ellipsoid or globose and up to 5 cm across. Furthermore, from our examinations, we found the shape and size of leaves of this species can be elliptic, oblong-elliptic, ovate, elliptic-ovate or lanceolate-ovate and sometimes larger, 10–24 × 4–9.5 cm; the male inflorescences in clusters of 2–7 flowers; the staminodes are absent or present; the ovaries have 4–9 locules; and the shape and size of fruits can be broadly ellipsoid, subglobose, globose or depressed globose and sometimes larger, 1.8–5.5 × 2–5.6 cm, without or with a short, thick beak at the apex.

Nazre (2010: 1256) notes that no type specimen was mentioned by Wallich (Wallich, 1832) in his description of *Garciniaspeciosa* but appeared earlier in *Wallich Catalogue 4855* (Wallich, 1828–1849) collected from Amherst, Myanmar which Maheshwari (1964: 123) considered the specimen at CAL [without barcode] as the lectotype, and Nazre et al. (2018: 17) mentioned with an isolectotype at K-W [without barcode]. However, we located the lectotype at CAL [CAL0000065160] and isolectotype at K-W [K001104074].

In the original description of *Garciniabenthamii* by Pierre (1882: t. 55, 56.), only one specimen is cited, “*Herb. Pierre n° 70*” collected from Cambodia but he did not select a holotype nor did he mention the name of the herbarium where the specimen was housed, and following Art. 9.6 of the ICN (Turland et al. 2018), it constitutes a syntype. The name *G.benthamii* has been lectotypified by Nazre (2010: 1256), who designated the specimen *Pierre 700* collected from Cambodia, and deposited at P [P00329872], with isolectotypes at A [without barcode] and K [without barcode]. However, this specimen has a different collector number than that reported in the original publication of this name. According to Nazre (2010), type specimens of *G.benthamii* have multiple sheets taken from different localities in Cambodia and Vietnam where some were cultivated. However, based on the *Pierre 700* sheets that we examined from P [P00329872, P04701491] and L [U1208099, U1208248], they were all collected in Vietnam (ad Bung in prov. Saïgon). We have not seen isolectotypes at A and K. The collector number *Pierre 70* cited in Pierre (1882) appears to have been an error and should have been *700*.

*Garciniariedeliana* was named by Pierre (1883: 12. t. 79A), who stated only one specimen, *Riedel s.n.* collected from Gorontalo, Sulawesi (also known as Celebes). Nazre et al. (2018: 17) designated this material at K [without barcode] as the lectotype, with an isolectotype at P [without barcode]. However, we could locate the lectotype at K [K000380456] and we located three sheets of isolectotypes at P [P04700635, P04700639, P04700640].

*Garciniarumphii* was named by Pierre (1883: 13. t. 77A), who cited two specimens: *Treub 4168* and *Treub 4169* collected from Bangka Island, Indonesia. He did not mention the name of the herbarium where the materials were kept, and following Art. 9.6 of the ICN (Turland et al. 2018), they constitute syntypes. The name *G.rumphii* has been lectotypified in a first-step by Nazre (2010: 1256–1257) using the material *Treub 4169* at P [without barcode], and in a second-step by Nazre et al. (2018: 17) using the material *Treub 4169* at P [P04700302], with isolectotypes at P [P04700298, P04700299, P04700301]. However, from our examination of specimens, we found that the P [P04700301] sheet is labeled with *Treub 4168*. We also located one sheet of isolectotype at K [K000380451].

In the original publication of *Garciniakurzii* by Pierre (1883: 14. t. 78A.), only one gathering is stated, *Kurz 24* collected from Andamans. He did not mention the name of the herbarium in which it was present, and following Art. 9.6 of the ICN (Turland et al. 2018), it constitutes a syntype. Maheshwari (1964: 123) selected a specimen at CAL [without barcode] as the lectotype, without isolectotype. However, we could not locate the lectotype at CAL, and we could trace two sheets of isolectotypes at P [P00329890, P00329891].

Pierre (1883: 37) established *Garciniajawoera* based on the materials collected from Tandjoor, Java but he did not choose a holotype nor did he mention the name of the herbarium where the specimens were housed. This name has been lectotypified twice, first by Nazre (2010: 1257), who designated the material *Pierre 4607* at P [without barcode] collected from Tandjoor, Java, with isolectotypes at P [without barcode] and L [without barcode], and second time by Nazre et al. (2018: 17), who selected the same collection at P [P00379817], with isolectotypes L [without barcode] and P [P00379816, P00379818]. We have not seen the isolectotype at L.

Pierre (1883: 37) erected *Garciniakrawang* based on the specimen *Korthals 1313a* collected from Pulau Lampei (Lampei Island), South Kalimantan, Borneo. The name *G.krawang* has been lectotypified in a first-step by Nazre (2010: 1257) using the specimen *Korthals 1313a* at P [without barcode], and in a second-step by Nazre et al. (2018: 17) using the specimen *Korthals 1313a* (*Herb. Pierre 4601*) at P [P00379812], without isolectotype. However, we located an isolectotype at P [P00379813].

*Garciniakingii* was named by Pierre but unpublished, and then this name was described by Vesque (1893: 407) based on the specimen collected from Andaman Island. This name has been lectotypified twice, first by Nazre (2010: 1257), who selected the specimen collected by King (*King’s Collector s.n.*) from Andaman Island housed in K [without barcode], and then again by Nazre et al. (2018: 17), who designated the same specimen. The first lectotypification has priority. We viewed the lectotype at K [K000380453].

##### Additional specimens examined.

**Thailand. Northern.** Chiang Mai [Mae Kuang, near Doi Saket, female fl., 1 Mar 1910 (as *G.cornea*), *A. F. G. Kerr 1020* (BM, K); Doi Suthep, male fl., 27 Mar 1910 (as *G.cornea*), *A. F. G. Kerr 1073* (BM, K, L [L2408860]); Doi Suthep, fl., 11 Feb 1923 (as *G.cornea*), *Winit s.n.* (BK, BM); Suthep Subdistrict, male fl., 5 Mar 1937 (as *G.speciosa*), *J. Samutnavee 13/2481* (BKF); Doi Suthep, fr., 28 Apr 1958 (as *G.speciosa*), *T. SØrensen* et al. *3103* (BKF, C); Doi Suthep, 12 Jul 1958 (as *Garcinia* sp.), *T. SØrensen* et al. *4025* (C); Doi Suthep, 5 Oct 1958 (as *Garcinia* sp.), *T. SØrensen* et al. *5460* (C); Doi Suthep, 19 Feb 1959 (as *G.speciosa*), *T. SØrensen* et al. *6958* (BKF, C); Montha Than Waterfall, Doi Suthep, sterile, 5 Feb 1983 (as *G.thorelii*), *W. Wattanadechseri 25205368* (QBG); Doi Suthep, male fl., 16 Feb 1988 (as *G.speciosa*), *J. F. Maxwell 88-190* (AAU, BKF, L [L2408855]); Doi Suthep-Pui National Park, sterile, 23 Apr 2003 (as *G.speciosa*), *J. F. Maxwell* et al. *4* (CMUB); Mae Khan, male fl., Mar 1913 (as *G.cornea*), *Winit 70* (BM, K); Doi Inthanon, fr., 9 May 1958 (as *G.speciosa*), *T. SØrensen* et al. *3334* (BKF, C); Wachirathan Waterfall, Doi Inthanon, male fl., 27 Feb 1979 (as *G.speciosa*), *H. Koyama* et al. *15566* (AAU, BKF); Doi Inthanon National Park, sterile, 21 Jul 1988 (as G.cf.vilersiana), *C. Phengklai* et al. *6708* (BKF); Ban Mae Bon, Phrao District, fr., 12 Jul 1996 (as G.cf.speciosa), *BGO Staff 6823* (QBG); Ban Kio Lom, Bo Luang Subdistrict, Hot District, male fl., 18 Mar 2003 (as *Garcinia* sp.), *T. Wongprasert 033-53* (BKF); Forest Fire Control Station, Doi Inthanon National Park, fr., 18 May 2003 (as *Garcinia* sp.), *T. Wongprasert & S. Khaoiam 035-25* (BKF)]; Chiang Rai [Doi Duan, male fl., 19 Mar 1921 (as *G.speciosa*), *A. F. G. Kerr 5107* (BM, K, P [P04899657]); Mae Fang, fl., 3 Mar 1928 (as *Garcinia* sp.), *Winit 1876* (BK, K)]; Phayao [Doi Luang National Park, Mueang Phayao District, fl., 10 Feb 2016 [as *G.propinqua*], *N. Muangyen 717* (QBG)]; Phrae [Mae Yuak, male fl., 3 Mar 1911 (as *G.speciosa*), *Luang Vanpruk 237* (BKF)]; Phitsanulok [Thung Salaeng Luang National Park, fr., 20 Jun1967 (as *Garcinia* sp.), *S. Phusomsaeng 243* (BKF); Lan Hin Taek, Phu Hin Rong Kla National Park, Nakhon Thai District, female fl., 24 Feb 2007, *C. Ngernsaengsaruay G52-24022007* (BKF, spirit material)]; **North-Eastern.** Loei [Phu Kradueng, Wang Saphung District, male fl., 6 Mar 1942 (as *G.speciosa*), *Amporn 128* (BKF); Phu Kradueng, male fl., 6 Mar 1942 (as *G.speciosa*), *Warison 128* (BKF 2834); Phu Kradueng, male fl., 6 Mar 1946 (as *Garcinia* sp.), *Nat 214* (P [P05062030]); Phu Kradueng, fl., 13 Mar 1948 (as *G.speciosa*), *K. Suvatabundhu 83*, *84* (BK); Phu Kradueng, fr., 20 Apr 1955 (as *G.speciosa*), *T. Smitinand 2483* (BKF); near Huai Phai Waterfall, Phu Ruea National Park, male fl., 4 Mar 1993 (as *Garcinia* sp.), *P. Chantaranothai* et al. *1034* (BKF); Na Haeo, young fr., 26 Apr 1994 (as *G.cowa*), *W. Nanakorn* et al. (*BGO. Staff*) *3186* (AAU, QBG); en route from Khok Nok Kraba to Lon Tae, Phu Luang Wildlife Sanctuary, very young fr., 14 May 1998 (as *Garcinia* sp.), *T. Wongprasert* et al. *s.n.* (BKF 123962); Phu Luang Wildlife Sanctuary, very young fr., 14 May 1998 (as *Garcinia* sp.), *K. Chayamarit* et al. *1398* (BKF); Huai Baeng Forest Protection Station, Phu Luang Wildlife Sanctuary, fr., 22 Jun 2003 (as *Garcinia* sp.), *T. Wongprasert 036-46* (BKF); Phu Luang Wildlife Sanctuary, fr., 11 Jun 2023, *C. Ngernsaengsaruay G53-11062023* (BKF); Phu Ruea National Park, fr., 23 Mar 2004 (as *Garcinia* sp.), *S. Bunwong* et al. *267* (AAU); Phu Ruea District, fr., 23 Jul 2007 (as *Garcinia* sp.), *T. Wongprasert 077-31* (BKF)]; Nong Khai [Phon Phisai District, male fl., 25 Feb 1924 (as *G.speciosa*), *A. F. G. Kerr 8572*, *8572A* (BK, BM, K)]; Bueng Kan [Mueang Bueng Kan District (formerly Chaiyaburi), fl., 20 Feb 1924 (as *G.speciosa*), *A. F. G. Kerr 8513* (BM, K); Chet Si Waterfall, Seka District, male fl., 25 Feb 2003 (as *Garcinia* sp.), *T. Wongprasert 032-30* (BKF)]; Sakon Nakhon [Phu Phan National Park, male fl., 9 Mar 1996 (as *G.hombroniana*), *P. Puudjaa* 194 (BKF)]; Khon Kaen [Locality not specified, female fl. and young fr., 20 Mar 1942 (as *G.speciosa*), *Jirapha 36* (BKF 8457)]; **Eastern.** Chaiyaphum [Phu Khiao, male fl., 25 Feb 1931 (as *G.speciosa*), *A. F. G. Kerr 20261* (BK, BM, K); Phu Khiao, fr., 3 Aug 1972 (as *Garcnia* sp.), *K. Larsen* et al. *31355* (AAU); Ban Nam Phrom, young fr., 24 May 1974 (as *G.speciosa*), *R. Geesink* et al. *6922* (AAU, BKF, C, K, L [L 0089486], P [P05061691]); Tat Ton Waterfall, Tat Ton National Park, fr., 19 Jun 2003 (as *Garcnia* sp.), *T. Wongprasert 036-3* (BKF)]; Ubon Ratchathani [Huai Phok Waterfall, Dong Na Tham Forest, Pha Taem National Park, Khong Chiam District, fr., 1 Mar 2007 (as *G.cowa*), *S. Suddee* et al. *3082* (BKF)]; **South-Western.** Uthai Thani [Ban Rai District, fr., 17 Nov 1961 (as *Garcnia* sp.), *B. Sangkhachand 250* (AAU, C, K); Huai Kha Khaeng Wildlife Sanctuary, Ban Rai District, male fl., 20 Feb 1970 (as G.cf.hombroniana), *C. F. van Beusekom & T. Santisuk 2866* (AAU, BKF, C, E [E00839762], P [P05062059]); ibid., female fl., 20 Feb 1970 (as G.cf.hombroniana), *C. F. van Beusekom & T. Santisuk 2879* (AAU, BKF, C, E [E00839761], P [P05062010]); ibid., male fl., 22 Feb 1970 (as G.cf.hombroniana), *C. F. van Beusekom & T. Santisuk 2916* (AAU, BKF); Huai Kha Khaeng Wildlife Sanctuary, fr., 10 Apr 1996 (as *Garcnia* sp.), *T. Wongprasert* et al. *s.n.* (BKF 109898)]; Kanchanaburi [Ban Cha Kae Yai, male fl., 28 Feb 1973 (as *Garcnia* sp.), *C. Phengklai* et al. *3069* (BKF, C, K, L [L2409478], P [P05062048]); Than Thong Waterfall trail, Chaloem Rattanakosin National Park, Si Sawat District, sterile, 28 Mar 2018 (as *G.mangostana*), *W. La-ongsri* et al. *5577* (QBG)]; Phetchaburi [Kaeng Krachan National Park, sterile, 8 Mar 1994 (as *G.hombroniana*), *T. Santisuk* et al. *s.n.* (BKF); Kaeng Krachan National Park, male fl., 29 Jan 2005 (as *Garcnia* sp.), *K. Williams* et al. *1189* (BKF)]; Prachuap Khiri Khan [Bang Saphan Yai, Bang Saphan District. male fl., 13 Nov 1944 (as *G.hombroniana*), *Taew 117* (BKF); Huai Yang National Park, Thap Sakae District, male fl., 26 Jan 2004 (as *Garcnia* sp.), *D. J. Middleton* et al. *2509* (A [00466332], BKF)]; **Central.** Saraburi [Phu Khae Botanical Garden, 23 Apr 2017, *C. Ngernsaengsaruay* own observation] **South-Eastern.** Sa Kaeo [Nong I Lom, sterile, 15 Mar 1932 (as *G.speciosa*), *Unkonwn 82* (BKF 209)]; Prachin Buri [Yan Ri Subdistrict, Kabin Buri District, sterile, 13 Jun 1936 (as *G.speciosa*), *S. Arirop s.n.* (BKF)]; Chon Buri [Si Racha District, fr., 14 Apr 1922 (as *G.speciosa*), *D. J. Collins 788* (BK, K); Si Racha District, fl. buds, 23 Apr 1923 (as *G.speciosa*), *D. J. Collins 898* (BK, K, L [L0535229, L2408870]); Nong Nok Takrum, near Si Racha District, male fl., 3 Nov 1927 (as G.cf.cornea), *D. J. Collins 1693* (BK, K, L [L2408856]); Si Racha District, young fr., 19 Dec1927 (as G.cf.speciosa), *D. J. Collins 1823* (BK, BM, K); Si Racha District, male fl., Nov 1934 (as *G.speciosa*), *D. J. Collins s.n.* (K, P [P04701267]); Ko Khram, Sattahip District, male fl., 22 Oct 1999 (as *G.speciosa*), *C. Phengklai* et al. *12064* (BKF); Khao Khiao Open Zoo, very young fr., 10 Dec 2000 (as *G.speciosa*), *C. Phengklai* et al. *12847* (BKF)]; Chanthaburi [Khlung District, young fr., 3 Dec 1924 (as G.cf.speciosa), *A. F. G. Kerr 9526* (BK, BM, K); Khao Khitchakut National Park, fr., 14 Apr 1925 (as *G.speciosa*), *Nai Noe 71* (BK, BM, K); Ban Phluang, fl., 24 Nov 1930 (as G.cf.speciosa), *M. C. Lakshnakara 522* (BK, C, K, L [L2408869], P [P04701266]); Pong Nam Ron District, male fl., 19 Feb 1956 (as *Garcnia* sp.), *B. Sangkhachand 600* (C, P [P05062028]); Makham District, fr., 12 Jan 1958 (as *Garcnia* sp.), *T. SØrensen* et al. *162*, *163* (C); Khao Khitchakut National Park, fr., 8 Feb 1987 (as *G.hombroniana*), *C. Niyomdham* et al. *1319* (AAU, BKF, C, K); Trat [Bo Rai District, male fl., 27 Nov 1924 (as *G.speciosa*), *A. F. G. Kerr 9451* (BM, K); Dan Chumphon, fr., 19 Dec 1929 (as G.cf.speciosa), *A. F. G. Kerr 17613* (BK, BM, K); Khao Kuap, fl., 26 Dec 1929 (*Garcinia* sp.), *A. F. G. Kerr 17784* (BK); Ko Chang, fr., 22 Feb 1955 (as *G.hombroniana*), *T. Smitinand 2274* (BKF); Than Mayom Waterfall, Ko Chang, fr., 12 Mar 1970 (as *Garcinia* sp.), *C. F. van Beusekom & T. Santisuk 3195* (AAU, BKF, C, L [L0089540, L2409572, L2409573], P [P04700768]); Ao Salat, Ko Kut, fr., 5 Apr 1959 (as *G.hombroniana*), *T. Smitinand 5678* (BKF); Ko Kut, fr., 5 Apr 1959 (as *Garcinia* sp.), *T. SØrensen* et al. *7177* (BKF, C); Ko Kut, female fl. and young fr., 20 Oct 2000 (as *G.speciosa*), *C. Phengklai* et al. *13092* (BKF); Khlong Chao, Ao Phrao, Ko Kut [Leaves belong to *G.celebica* but fruits belong to *G.cowa*], 7 Apr 2002 (as *G.speciosa*), *C. Phengklai* et al. *13468* (BKF); Khao Lan, Khlong Yai District, fr., 24 Feb 2018, *C. Ngernsaengsaruay G53-24022018* (BKF); Ko Kut, 15 Oct 2022, *C. Ngernsaengsaruay* own observation]; **Peninsular.** Chumphon [Ban Thung Kha, young fr., 13 Jan 1927 (as G.cf.speciosa), *A. F. G. Kerr 11412* (BK, BM, K); Sand dune, Pathio District, 24 Apr 2022, *C. Ngernsaengsaruay* own observation]; Ranong [Ko Boi Noi, fr., 22 Feb 1966 (as *G.hombroniana*), *Sakol Sutheesorn 902* (BK)]; Surat Thani [Ko Tao, fr., 30 Dec 1926 (as *G.speciosa*), *A. F. G. Kerr 11179* (BM, C, K, L [L2408868], P [P04701273]); Ko Tao, male fl., 15 Apr 1927 (as G.cf.cornea), *A. F. G. Kerr 12752* (BM, K, L [L2408857]); Tha Khanon Subdistict, fl., 28 Aug 1931 (as *G.speciosa*), *Luang Saman 45* (BKF); Tha Chang District, fl., 15 Jan 1935 (as *G.hombroniana*), *Luang Saman 2585* (C, SING); Khao Tok Nong, Thung Thong Non-hunting Area, Khiansa District, fl., 23 Apr 2005 (as *Garcnia* sp.), *R. Pooma* et al. *5172* (AAU, BKF); Ko Pha Luai, male fl., 21 Apr 2009 (as *G.cowa*), *C. Phengklai* et al. *15862* (BKF); Khao Ra, Than Sadet-Ko Pha-Ngan National Park, 17 Sep 2017, *C. Ngernsaengsaruay* own observation]; Phangnga [Ko Yao Yai, fr., 4 Mar 1929 (as G.cf.speciosa), *A. F. G. Kerr 17338* (BK, BM, K); Ko Boi Noi, fr., 22 Feb 1966 (as *G.hombroniana*), *B. Hansen & T. Smitinand 12429* (BKF, E [E00839760]); Ko Miang, Mu Ko Similan National Park, fl., 15 Jan 1992 (as *Garcnia* sp.), *C. Niyomdham 2904* (AAU, BKF); Ko Yao, fr., 2 Apr 1998 (as *Garcnia* sp.), *P. Triboun & M. Triboun 922* (BK); Ko Yao Yai, sterile, 30 Apr 2007 (as *G.mangostana*), *C. Phengklai* et al. *15518* (BKF)]; Krabi [Khao Pra Bang Khram, fr., 4 Apr 1988 (as *G.hombroniana*), *C. Niyomdham & W. Ueachirakan 1762* (AAU, BKF, K); Khao Pra Bang Khram Wildlife Sanctuary, Khlong Thom Nuea Subdistrict, Khlong Thom District, fr., 26 Mar 2006 (as *G.hombroniana*), *J. F. Maxwell 06-205* (CMUB, QBG); ibid. fr., 15 Feb 2022, *C. Ngernsaengsaruay* et al. *G54-15022022* (BKF, spirit material); Ao Nang, fr., s.d. (as *Garcnia* sp.), *K. Larsen* et al. *43371* (AAU); Ko Lanta, female fl., 5 May 2013 (as *Garcnia* sp.), *B. Sonsupab L-50* (BK)]; Nakhon Si Thammarat [Khiriwong, sterile, 1 Sep 1952 (as *G.hombroniana*), *P. Suvarnakoses 423* (BKF); Karome Waterfall, Khao Luang National Park, Lansaka District, fr., 17 Mar 1985 (as *G.hombroniana*), *J. F. Maxwell 85-300* (A [00466348], AAU, BKF, PSU); Krung Ching Waterfall, Khao Luang National Park, Tha Sala District, fr., 14 Mar 2005 (as *Garcinia* sp.), *S. Gardner* et al. *ST1671* (BKF, K); ibid., female fl., 27 Feb 2006 (as *Garcinia* sp.), *S. Gardner & P. Sidisunthorn ST1671a* (K)]; Trang [Kachong, sterile, 9 Jun 1933 (as *G.speciosa*), *Put 271* (BKF); Khao Chong, male fl., 15 Jun 1966 (as *G.speciosa*), *C. Boonnab & L. Phuphathanaphong 293* (BKF); Ton Te Waterfall, Palian District, fr., 2003 (as *G.speciosa*), *A. Sinbumroong & S. Davies AS405* (BKF); Locality not specified, male fl., 14 Jan 1916 (as *G.cornea*), *Luang Vanpruk 816* (K)]; Satun [Ko Adang, Tarutao National Park, fl., 14 Jan 1928 (as *G.cornea*), *A. F. G. Kerr 14078* (BK, BM, K, L [L2408859]); Ko Tarutao, fl., 20 Jan 1928 (as *G.cornea*), *A. F. G. Kerr 14225* (BK, K); Ko Tarutao, male fl., 11 Nov 1979 (as *G.hombroniana*), *G. Congdon 148* (AAU, PSU); Ko Tarutao, fl., 14 Nov 1979 (as *G.hombroniana*), *G. Congdon 165* (AAU, PSU); Ao Phante, Ko Tarutao, sterile, 30 Jul 1980 (as G.cf.hombroniana), *G. Congdon 801* (A [00466349], AAU, PSU); Ao Son, Tarutao National Park, La Ngu District, fr., 10 Feb 2005 (as *Garcinia* sp.), *P. Sidisunthorn & P. Tippayasri ST1488* (K); road to Ao Son, Tarutao National Park, La Ngu District, fl., 11 Feb 2005 (as *Garcinia* sp.), *S. Gardner ST1501* (K); Ao Russi, Tarutao National Park, La Ngu District, sterile, 21 May 2005 (as *Garcinia* sp.), *S. Gardner* et al. *ST1859* (K); ibid., fr., 21 May 2005 (as *Garcinia* sp.), *Gardner* et al. *ST1860* (K); Tarutao National Park, La Ngu District, fl., 1 Apr 2006 (as *Garcinia* sp.), *P. Sidisunthorn ST2535* (K); Ko Tarutao, young fr., 8 Apr 2008 (as *G.mangostana*), *C. Phengklai* et al. *15703* (BKF); Ko Tarutao, fr., 8 Apr 2008 (as *G.mangostana*), *C. Phengklai* et al. *15806* (BKF); Ko Tarutao, 8 Apr 2008 (as *Garcinia* sp.), *B. Sonsupab 3981* (BK)]; Songkhla [Sadao District, fl., 22 Feb 1941 (as *G.hombroniana*), *T. Premrasami s.n.* (BKF); Khlong Huai Phlu, Prik Subdistrict, Sadao District, sterile, 12 Mar 1954 (as *G.speciosa*), *Snguan s.n.* (BKF); Khao Noi, fl., Feb 1950 (as *Garcnia* sp.), *L. Williams 17272* (K); Khao Noi, female fl., s.d. (as *G.hombroniana*), *T. Smitinand & Williams 17272* (BKF); Ton Nga Chang Wildlife Sanctuary, fr., 10 May 1979 (as *G.hombroniana*), *H & C 444* (PSU); Ton Nga Chang Waterfall level 5, Hat Yai District, fl., 14 May 2004 (as *Garcinia* sp.), *S. Gardner ST0524* (K); Ton Nga Chang Waterfall level 3, Hat Yai District, fl., 30 Jan 2006 (as *Garcinia* sp.), *S. Gardner ST2278* (K); Boriphat Waterfall Park, Rattaphum District, male fl., 8 Feb 1985 (as *G.hombroniana*), *J. F. Maxwell 85-167* (BKF, PSU); Boriphat Waterfall, fl., 17 Dec 2003 (as *Garcnia* sp.), *A. S. Barfod* et al. *583* (AAU); Prince of Songkhla University, Hat Yai District, fr., 29 Apr 1985 (as *G.hombroniana*), *P. Sirirugsa 1013* (BKF, PSU); Khao Kho Hong, Prince of Songkhla University, Hat Yai District, fr., 24 Mar 2008 (as *G.hombroniana*), *N. Boonnak 006*, *007* (PSU); Khlong Rhang Hill, Na Mom District, fr., 15 May 1985 (as *G.hombroniana*), *J. F. Maxwell 85-474* (AAU, BKF, PSU); Khlong Rhang Hill, Na Mom District, male fl., 15 Feb 1986 (as *G.hombroniana*), *J. F. Maxwell 86-68* (AAU, BKF, PSU); Khao Tang Kuan, fl., 30 Aug 1997 (as *G.hombroniana*), *N. Yutaworawit 2E* (PSU); Ban Taling Chan, Chana District, fr., 17 May 1999 (as *G.maingayi*), *S. Petchsri 1* (PSU)]; Pattani [Nong Chik District, fr., 21 Jul 1990 (as *G.hombroniana*), *T. Santisuk s.n.* (BKF)]; Yala [Betong District, fl., 24 Feb 1941 (as *G.hombroniana*), *T. Premrasami 129* (BKF)]; Narathiwat [Waeng District, fr., 6 Sep 1966 (as *G.robusta*), *B. Sangkhachand & B. Nimanong 1320* (BKF); Waeng District, fr., 8 Sep 1966 (as *Garcinia* sp.), *Prayad 373* (BK); Khao Tan Yong, sterile, 5 Jun 1973 (as *Garcinia* sp.), *C. Chai-anan 451* (BKF); Forest behind Wat To Mo, Sukhirin District, fr., 9 Aug 1996 (as *Garcinia* sp.), *P. Puudjaa 264* (BKF)].

#### ﻿2. Garcinia exigua

Taxon classificationPlantaeMalpighialesClusiaceae

Nazre, Phytotaxa 373(1): 28. figs 2g, 3i & 10. 2018.; Ngerns. et al., Thai Forest Bull., Bot. 51(1): 36–44. figs 1–3. 2023.

44E904B9-92FE-5EDC-BE12-83C914BDA727

Fig. 4

##### Type.

Malaysia, Borneo, Sarawak, Bintulu, Buan Forest Reserve, 18 Sep 1972, *P. Chai S31750* (holotype SAR, reported by Nazre et al. 2018, not seen; isotype L [L2403372, photo seen]).

##### Description.

***Habit*** trees, 2.5–20 m tall, 20–100 cm GBH, sometimes with buttresses near the base of the main stem of large trees; latex yellow, sticky; branchlets green, 4-angular, glabrous. ***Bark*** brown or dark brown, after the peel pale yellow or pale brown, mottled, flaking and leaving roundish or irregularly shaped scars; inner bark red. ***Leaves***: lamina narrowly elliptic, elliptic, broadly elliptic, sometimes obovate, 3–6.3 × 1–3.2 cm, apex narrowly obtuse or obtuse, base cuneate, margin entire and finely revolute, coriaceous, smooth, shiny dark green above, paler below, glabrous on both surfaces, midrib raised on both surfaces, secondary veins 10–18 each side, curving towards the margin and connected in distinct loops and united into an intramarginal vein, faint above, inconspicuous below, with intersecondary veins, veinlets reticulate, faint on both surfaces, with a few scattered black gland dots on both surfaces, interrupted long wavy lines of differing lengths, nearly parallel to the midrib, running across the secondary veins to the apex or the margin, visible on both surfaces especially on the lower surface of dry leaves; petiole green, 0.3–1 cm long, 1–1.5 mm in diam., grooved above, finely transversely rugose, glabrous, with a basal appendage clasping the branchlet; young leaves pale green, glossy; fresh leaves brittle when crushed; dry leaves pale brown or reddish brown. ***Inflorescences*** terminal, in fascicles of 3 male flowers and usually solitary in female flowers (observations based on infructescence); bracts 2, caducous, green or brownish green, conduplicate with a central keel, ovate, 8–10 × 4–5.5 mm, apex acuminate, thinly coriaceous (of male inflorescences). ***Flowers***: sepals and petals glabrous. ***Male flower buds*** subglobose to globose, 3.5–5.5 mm in diam. ***Male flowers*** lightly fragrant, 1.5–2 cm in diam., the middle flowers always largest; bracteoles caducous; pedicel pale green, turning pale yellow, 3–4.5 mm long, 1.3–1.7 mm in diam., glabrous; sepals 4, pale yellow, concave, thinly coriaceous, with wavy lines outside, the outer pair broadly ovate, 4.2–5 × 3.5–5 mm, apex acute, the inner pair elliptic or broadly elliptic, 4.2–6 × 3–4.5 mm, apex obtuse; petals 4, pale yellow, elliptic, 6.5–9 × 3.5–8 mm, subequal, apex obtuse, margin revolute, thinly coriaceous, with wavy lines outside; stamens numerous, united into a single 4-lobed bundle, surrounding a pistillode, lobes 3–3.5 × 3–4 mm; filaments 0.5–0.7 mm; anthers 0.9–1.2 × 0.8–1 mm; pistillode fungiform, 3–3.5 mm long; sterile stigma yellow, sessile, convex, weakly 4-lobed, 2.5–3 mm in diam., smooth. ***Female flowers*** not seen. ***Fruits*** green, smooth with fine longitudinal striate, glabrous, with a sticky yellow latex, subglobose, globose or broadly ellipsoid, 1–1.3 × 0.8–1.1 cm, pericarp coriaceous; persistent stigma dark brown or blackish brown, flattened or slightly convex, 2–3 mm in diam., weakly 4-lobed; persistent sepals green, concave, coriaceous, lanceolate-ovate or ovate, 3.5–5.5 × 3–5 mm, the outer pair slightly smaller than the inner pair, apex acute; fruiting stalk green, 1–2 mm long, 1–1.8 mm in diam., glabrous. ***Seeds*** 1, brown (dark brown when dry) mottled with irregular lines, ellipsoid, c. 6 × c. 3.5 mm, c. 1.8 mm thick, compressed, rounded at both ends, with a thin fleshy pulp. The morphological characters and data reported here for this species were mostly taken from Ngernsaengsaruay et al. (2023a).

**Figure 4.**
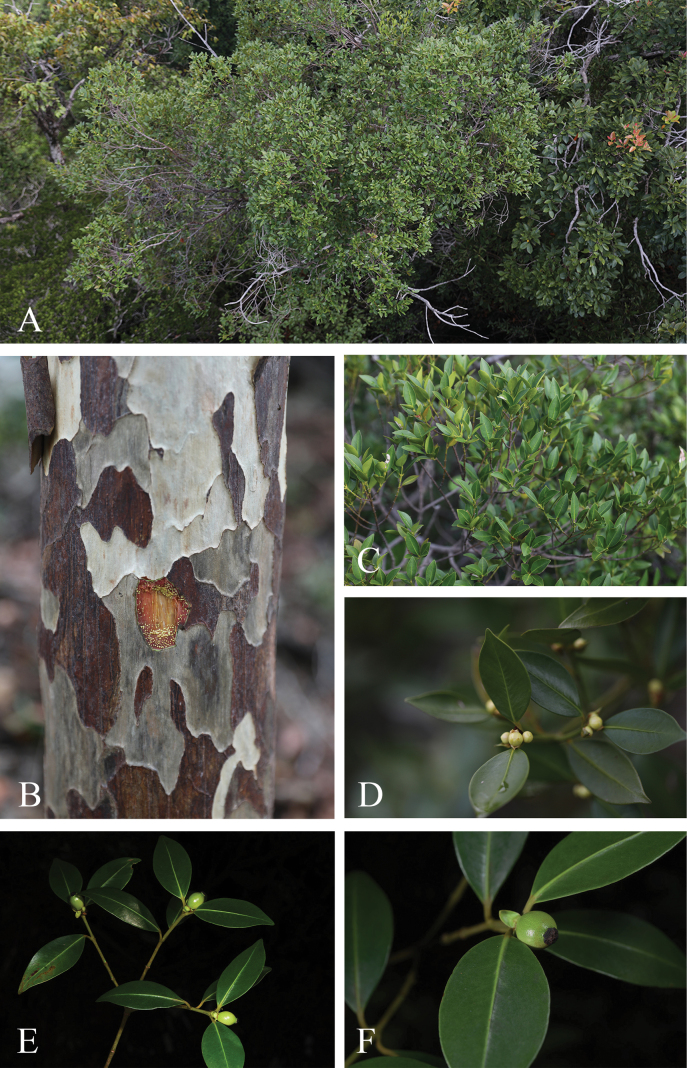
*Garciniaexigua***A** habitat and canopy (top view) **B** stem, bark, and slashed bark with yellow latex **C** branchlets and leaves **D** branchlets and male inflorescences with male flower buds **E, F** branchlets, leaves, and young fruits. Photos: Chatchai Ngernsaengsaruay (**A–D**), Naiyana Tetsana (**E, F**).

##### Distribution.

Thailand, Malaysia [Borneo, Sarawak (Bintulu, Buan Forest Reserve; Mulu National Park: Matong Ubong, Ulu Matong, Sungai Ubong)]; Brunei [Temburong (Ulu Belalong)].

##### Distribution in Thailand.

**Peninsular**: Krabi.

##### Habitat and ecology.

It is found in dry evergreen forest on limestone hills and littoral dry evergreen forest on limestone hills, 50–100 m amsl.

##### Phenology.

Flowering January to March; fruiting April to June.

##### Conservation status.

*Garciniaexigua* is a rare species in Borneo, and is represented only from three localities in lowland and hill forest in Sarawak and Brunei (Nazre et al. 2018). In Thailand, the species is known only from two localities in Krabi Province, but to be expected in other limestone hills. Globally, it is known only from Borneo to Thailand, and has an Extent of Occurrence (EOO of 51,767.17 km^2^) and a relatively small Area of Occupancy (AOO of 16 km^2^) which lies within protected and non-protected areas. It is inferred to be experiencing a continuing decline in habitat area, extent, and quality. We therefore consider the conservation assessment as Vulnerable [VU B2ab(iii)].

##### Etymology.

The specific epithet of *Garciniaexigua* is a Latin word, referring to the small size of all parts in the specimens (Nazre et al. 2018).

##### Vernacular names.

**Phawa bai lek krabi** (พะวาใบเล็กกระบี่) (Ngernsaengsaruay et al. 2023a); Kandis (Sarawak) from the material *Runi S. Pungga & P. C. Yii S61132* (K, L [L3811193], as *Garciniasarawhensis* Pierre).

##### Uses.

Not known.

##### Notes.

*Garciniaexigua* is recognized by the following characters: (1) The leaves and fruits are small. (2) The fresh leaves are brittle when crushed. (3) The stamens of the male flowers are united into a single 4-lobed bundle, surrounding a pistillode, and (4) The bark is mottled, flaking and leaving roundish or irregularly shaped scars similar to the bark of some species of *Lagerstroemia* L. in the Lythraceae, e.g., *L.duperreana* Pierre ex Gagnep., *L.floribunda* Jack and some species of *Terminalia* L. in the Combretaceae, e.g., *T.corticosa* Pierre ex Laness.

According to Nazre et al. (2018), the shape and size of leaves of *Garciniaexigua* are elliptic and 0.8–2.1 × 0.3–1 cm; however, from our observations, we found the leaves can be narrowly elliptic to broadly elliptic, sometimes obovate, and larger, 3–6.3 × 1–3.2 cm.

As mentioned by Nazre et al. (2018), the shape, size, and color of fruits of *Garciniaexigua* are globose or ellipsoid, 9.5–10.5 × 8.5–11 mm, and shiny green turning yellow when ripe; however, in this study, we found the fruits can be subglobose, globose or broadly ellipsoid, sometimes slightly longer, 1–1.3 × 0.8–1.1 cm, and green. However, we did not observe ripe fruits.

##### Additional specimens examined.

**Thailand. Peninsular**: Krabi [Wat Tham Suea (originally “Tham Sue” on the label), Mueang Krabi District, fr., 8 May 2002 (as *Garcinia* sp.), *P. Pooma* et al. *3612* (BKF, QBG); Ko Hong, trail up to view point, Than Bok Khorani National Park, Mueang Krabi District, male fl., 13 Feb 2022, *C. Ngernsaengsaruay* et al. *G27-13022022* (BKF, K, QBG); ibid., male fl., 13 Feb 2022, *C. Ngernsaengsaruay* et al. *G28-13022022* (BKF, K, QBG)].

**Malaysia. Borneo**: Sarawak [Mulu National Park, Sg. Matong Ubong, fr., 11 Nov 1990, *P. C. Yii & Runi S. Pungga S57293* (L [L3810871], SAR [reported by Nazre et al. 2018], as *Garciniasarawhensis*); Mulu National Park, Ulu Matong, fr., 13 Nov 1990, *P. C. Yii & Runi S. Pungga S60529* (BKF, L [L3811190], as *G.sarawhensis*); Mulu National Park, Sungai Ubong, fr., 19 Nov 1990, *Runi S. Pungga & P. C. Yii S61132* (K, L [L3811193], SAR [reported by Nazre et al. 2018], *as G.sarawhensis*). **Brunei.** Temburong [Ulu Belalong, 22 Jan 1994, *Coode* et al. *7886* (A [reported by Nazre et al. 2018]).

#### ﻿3. Garcinia mangostana

Taxon classificationPlantaeMalpighialesClusiaceae

L., Sp. Pl. 1: 443. 1753; Roxb. in Carey, Fl. Ind. 2: 619. 1832; Miq. Fl. Ned. Ind. 1(2): 506. 1859; Planch. & Triana, Ann. Sci. Nat., Bot., sér. 4, 14: 325. 1860; Laness., Mém. Gen. Garc.: 15. 1872; T. Anderson in Hook. f., Fl. Brit. India 1(2): 260. 1874; Kurz, J. Asiat. Soc. Bengal, Pt. 2, Nat. Hist. 43(2): 86. 1874 et Forest Fl. Burma 1: 87. 1877; Pierre, Fl. Forest. Cochinch. 1(4): t. 54. 1882; Vesque, Epharmosis 2: 17. t. 160, 161. 1889; King, J. Asiat. Soc. Bengal, Pt. 2, Nat. Hist. 59(2): 156. 1890; Vesque in A. DC. & C. DC., Monogr. Phan. 8: 386. 1893; Engl. in Engl. & Prantl, Die Naturlichen Pflanzenfamilien 3(6): 235. fig. 114 A, B. 1893; Brandis, Indian Trees: 49. 1906; Merr., Philipp. J. Sci. 3: 364. 1908; Gamble, Fl. Madras 1: 73. 1915; Pit. in Lecomte et al., Fl. Indo-Chine 1(4): 307. 1910; Ridl., Fl. Malay Penins. 1: 172. 1922; Merr., Enum. Philipp. Fl. Pl. 3: 85. 1923; C. E. Parkinson, Forest Fl. Andaman Isl.: 88. 1923; Corner, Wayside Trees Mal. 1: 318. ed. 2. 1952; Pételot, Arch. Rech. Agron. Cambodge Laos Vietnam 1: 62. 1952; Backer & Bakh. f., Fl. Java (Spermatoph.) 1: 387. 1963; Maheshw., Bull. Bot. Surv. India 6: 120. t. 2. fig. 14. 1964; Corner & Watan., Ill. Guide Trop. Pl.: t. 191. fig. 8. 1969; Whitmore in Whitmore, Tree Fl. Malaya 2: 215. 1973; D’Arcy, Ann. Missouri Bot. Gard. 67: 998. fig. 4B. 1980; S. W. Jones, Morphology and Major Taxonomy of Garcinia (Guttiferae), Ph.D. Thesis (unpublished): 288. 1980; Kosterm. in Dassan. & F. R. Forsberg, Revis. Handb. Fl. Ceylon 1: 88. 1980; H. Keng, Concise Fl. Singapore: 49. 1990; P. H. Hô, Câyco Vietnam 1: 559. fig. 1544. 1991; E. W. M. Verheij & R. E. Coronel (eds), PROSEA 2: 177, t. 178. 1992; N. P. Singh in B. D. Sharma & Sanjappa, Fl. Ind. 3: 143. 1993; X. W. Li, J. Li, N. Robson & P. F. Stevens in C. Y. Wu, P. H. Raven & D. Y. Hong, Fl. China 13: 43. 2007; W. E. Cooper, Austrobaileya 9(1): 17. 2013; S. Gardner, P. Sidisunthorn & Chayam., Forest Trees S. Thailand 1: 355. fig. 546. 2015. var. amangostana, Nazre et al., Phytotaxa 373(1): 31. fig. 12. 2018.

E02F6429-700E-5C22-AE56-52DA28BDA656

Fig. 5.

≡ Mangostanagarcinia Gaertn., Fruct. Sem. Pl. 2: 105. t. 105a–g. 1790.

##### Type.

illustration, “Mangoustan”, Garcin (1733: Philosop. Transact. 431. figs 1–9.) (lectotype, designated by Hammel in Jarvis et al. 1993: 28).

##### Description.

***Habit*** trees, 7–20(–25) m tall, 40–150 cm GBH; latex yellow, sticky; branchlets green, 4-ridged, glabrous. ***Bark*** dark brown, scaly; inner bark brownish orange. ***Leaves***: lamina elliptic, oblong-elliptic, oblong or ovate, 15.5–36 × 6.5–13 cm, apex acute or acuminate, base obtuse or oblique, sometimes cuneate, margin entire or repand, thickly coriaceous, smooth or slightly bullate, shiny dark green above, paler below, glabrous on both surfaces, midrib slightly raised above, raised as a prominent ridge below, secondary veins 10–18 each side, curving towards the margin and connected in distinct loops and united into 2 intramarginal veins, flattened above, raised and conspicuous below, intramarginal veins shallowly grooved above, with intersecondary veins, veinlets reticulate, visible below, interrupted long wavy lines of differing lengths, running across the secondary veins to the apex or the margin, conspicuous below; petiole green, stout, 1.5–2.7 cm long, 4–7 mm in diam., not grooved, distinctly transversely rugose, glabrous, with a basal appendage clasping the branchlets; young leaves brownish red or reddish brown, turning pale green, glossy; fresh leaves tough when crushed; mature leaves turning greenish yellow to pale yellow before falling off; dry leaves pale brown or reddish brown. ***Inflorescences*** terminal. ***Flowers***: sepals and petals glabrous. ***Male flowers*** not seen. ***Female flower buds*** subglobose to globose, 1–2 cm in diam. ***Female flowers*** solitary or in a cluster of 2–5(–7) flowers, 3.2–5 cm in diam.; bracteoles caducous; pedicel (of a flower in an inflorescence) or peduncle (of a solitary flower) green, stout, terete or slightly 4-angled, 1–2.4 cm long, 5.5–8 mm in diam., glabrous; sepals 4, pale green outside, bright red or yellowish red inside, concave, thickly coriaceous, suborbicular, orbicular or broadly elliptic, 1–2 × 1–2.2 cm, the outer pair slightly smaller than the inner pair, apex rounded; petals 4, yellowish red or yellowish pink, somewhat thick and fleshy, suborbicular, broadly elliptic, broadly obovate or broadly ovate, 1.1–2.1 × 1.4–2.6 cm, unequal, apex rounded, margin entire or irregularly lobed and undulated; staminodes 10–18, free, surrounding the ovary; filaments filiform, 2–5 × 0.5–1.2 mm, unequal; anthers pale yellow or brownish yellow, 1.2–1.7 × 1–1.4 mm; pistil fungiform, 0.6–1.2 cm long; ovary pale green, depressed globose or subglobose, 0.4–0.7 × 0.6–1.3 cm, glabrous, 4–8-locular; stigma pale yellow, convex, radiate, deeply 4–8-lobed, 1.5–3 mm long, 0.7–1.2 cm in diam., smooth. ***Fruits*** pale green or greenish pale yellow, turning pinkish pale yellow, pink, reddish purple to blackish purple when ripe, smooth, glabrous, with a sticky yellow latex, subglobose or globose, 3.4–6.2 × 3.8–7 cm, pericarp 0.4–1.2 cm thick, reddish purple, fleshy, becoming woody when dry; persistent stigma dark brown or blackish brown, flattened, radiate, deeply 4–8-lobed, 1.4–2.5 cm in diam., lobes wedge-shaped; persistent sepals green or green tinged with reddish purple, thickly coriaceous, 1.2–2.5 × 1.2–2.8 cm, usually larger than in flowering materials; fruiting stalk green, strong and thick, 1.2–2.6 cm long, 0.6–1.2 cm in diam., glabrous. ***Seeds*** 4–8, sometimes aborted, brown mottled with irregular lines, broadly ellipsoid, ellipsoid or semi-ellipsoid, 1.5–2.5 × 0.8–2 cm, compressed, rounded at both ends, with a white fleshy pulp.

**Figure 5.**
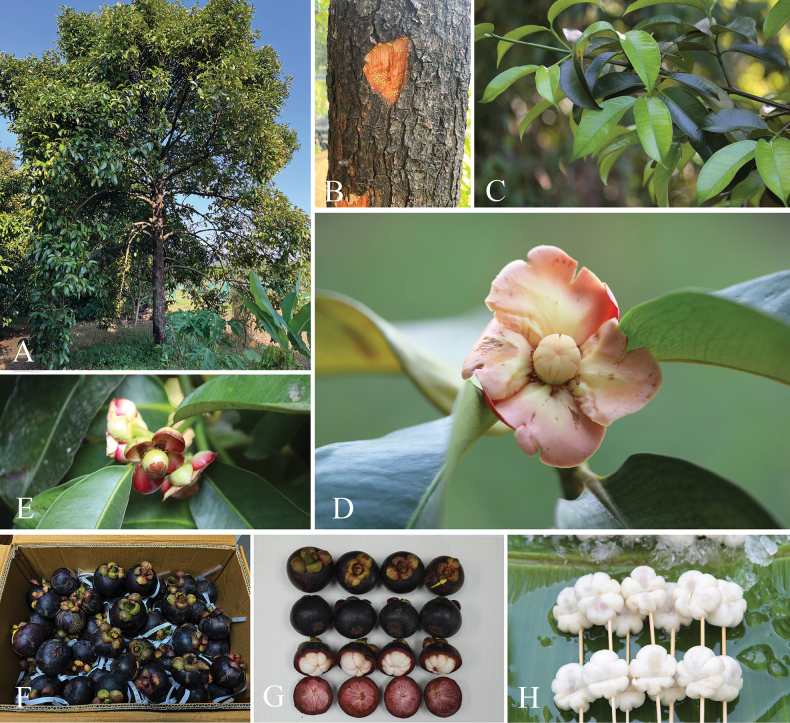
*Garciniamangostana***A** habit and habitat **B** stem, bark, and slashed bark with yellow latex **C** branchlets, young and mature leaves **D** branchlet and female flower **E** branchlet and female inflorescence with female flowers **F** ripe fruits **G** ripe fruits and seeds with a white fleshy pulp **H** seeds with a white fleshy pulp. Photos: Chatchai Ngernsaengsaruay.

##### Distribution.

The native range of this variety is Peninsular Malaysia. Cultivated throughout the tropics, mainly in Southeast Asia.

##### Distribution in Thailand.

It is cultivated throughout the country, especially in the peninsular and the south-eastern regions.

##### Habitat and ecology.

It is known only in cultivation. This species prefers humid climate.

##### Phenology.

In the south-eastern region: flowering December to February (March); fruiting February to June; harvesting April to May (June). In the peninsular region: flowering February to April; fruiting April to August; harvesting July to August [out-of-season: flowering August to October; fruiting October to February; harvesting December to February].

##### Conservation status.

Garciniamangostanavar.mangostana is widely cultivated throughout the tropics, especially in Southeast Asia. Because of its wide distribution, the number of localities, and because it is not facing any threat of extinction, we consider the conservation assessment as LC.

##### Etymology.

The specific epithet of *Garciniamangostana* is a Latin word, and is derived from the French “Mangoustan”, which translated to the English “Mangostan”, refers to mangosteen.

##### Vernacular names.

**Mangkhut** (มังคุด) (General); Măng cụt (Vietnam); Manggis (Malaysia, Indonesia, and Philippines); Manggustan (Philippines); Mangoustan, Mangoustanier (France); Mingut (Myanmar); Mongkhut (Cambodia); Mangosteen, Purple mangosteen (English).

##### Uses.

Mangosteen is widely cultivated as a fruit tree, especially in Southeast Asia. The juicy fleshy pulp surrounding the seeds is edible and has a sweet and sour taste. It is commonly known as the “queen of tropical fruits”. The juicy fleshy seed pulp can be used for making jams, beverages, ice creams, preserves (“Mang khut kuan” in Thai), and used fresh in syrup. In Nakhon Si Thammarat Province, the seeds with white fleshy pulp can be eaten raw (“Mang khut khat”) (Fig. 5H) or cooked, e.g., used for consumption in the southern Thai spicy sour yellow curries with fish or shrimp: “Kaeng Som” (sour curry) or “Kaeng Lueang” (yellow sour curry).

The fruit rind (pericarp) is used to tan leather and to produce black dye (Maheshwari 1964; Verheij and Coronel 1992). The wood is used for cabinetry, building purposes, rice pounders, and spear handles (Maheshwari 1964). It has been used in Thai traditional medicine for treatment of diarrhea and skin infections (Gritsanapan 1994). It contains tannins and xanthones, i.e., alpha-, beta- and gamma-mangostins (Govindachari et al. 1971; Jinsart et al. 1992; Nakatani et al. 2002). Alpha-mangostin is a major component which possesses anti-inflammatory (Chen et al. 2008) and antibacterial activities against methicillin-resistant *Staphylococcusaureus*, *S.epidermidis*, and *Propionibacteriumacnes* (Iinuma et al. 1996; Chomnawang et al. 2005). The fruit rind extract and mangostin have been known to possess antibacterial activity against bacteria causing acne. In Thailand, mangosteen fruit extract is popularly used as a food supplement while the fruit rind extract has been used in herbal cosmetics and pharmaceutical products. (Pothitirat and Gritsanapan 2008). The bark, young leaves, and fruit rind are used as a gargle for a sore mouth (Maheshwari 1964). In Thailand, the extract is popularly used in herbal cosmetics for anti-acne effect (Pothitirat and Gritsanapan 2008).

##### Notes.

*Garciniamangostana* was named by Linnaeus 1753: 443. It is the most important cultivated species in the genus of *Garcinia*. However, the latest taxonomic revision by Nazre et al. (2018) revealed that the species can be classified into three varieties: the cultivated variety G.mangostanavar.mangostana and two wild varieties G.mangostanavar.malaccensis (Hook. f.) Nazre and G.mangostanavar.borneensis Nazre. The varieties can only be distinguished with fertile and mature materials although male trees are rarely found.

Garciniamangostanavar.mangostana is very similar to G.mangostanavar.malaccensis and G.mangostanavar.borneensis and is distinguished by its male flowers with dwarf-fungiform (broadly fungiform) pistillodes, c. 5 mm long; fruits ovoid or globose; smooth stigma surface; and it is found only in cultivation. In contrast, the other two varieties have male flowers with small pistillodes c. 2 mm long or without pistillodes; globose, ellipsoid or ovoid fruits; rugose stigma surfaces; and they are found in the wild or in cultivation. G.mangostanavar.malaccensis differs from G. mangostanavar.borneensis in its stamens in a conical mass or slightly 4-angled, up to 1 cm long (vs in 4-angled, square-shaped, up to 6 mm long); fruits globose, ellipsoid or ovoid (vs globose); and stigma weakly to strongly raised (vs sessile). G.mangostanavar.malaccensis is found in lowland forests in Peninsular Malaysia, Singapore, Sumatra, and Borneo (Brunei and Sarawak), while G.mangostanavar.borneensis is found only in lowland forest of Borneo (East Coast of Sabah and Kalimantan) (Nazre et al. 2018).

As mentioned by Nazre et al. (2018), the shape and size of leaves of Garciniamangostanavar.mangostana are elliptic to broadly elliptic, ovate or oblanceolate and 9.2–25.5 × 7–9 cm; base of petiole without ligule-like appendage; however, from our observations, we found the leaves can be elliptic, oblong-elliptic, oblong or ovate, and sometimes larger, 15.5–36 × 6.5–13 cm; petiole with a basal appendage clasping the branchlets.

According to Nazre et al. (2018), the male flowers of Garciniamangostanavar.mangostana have a single square (4-angled) mass of stamens surrounding the base of the pistillode and up to 8 mm long. Based on our observations, the male plants have never been found in Thailand. Therefore, in Thailand mangosteen appear to be an obligately agamospermous species (apomictic species) with the production of seeds without fertilization.

As stated in Nazre et al. (2018), the shape and size of fruits of Garciniamangostanavar.mangostana are ovoid or globose and up to 6 cm across; however, from our examination of specimens, we found the fruits can be subglobose or globose, and sometimes larger, 3.4–6.2 × 3.8–7 cm.

##### Additional specimens examined.

**Thailand. Northern**: Chiang Mai [Doi Chiang Dao, fl., 28 Oct 1979 [as *Garcinia* sp.], cultivated, *T. Shimizu* et al. *T-20980* (AAU, BKF)]; **Central**: Suphan Buri [Si Samran Subdistrict, Song Phi Nong District, sterile, 9 Mar 2016, cultivated, *W. Sueksakit M10-1* (BK)]; Nakhon Pathom [Silpakorn University, sterile, 20 Apr 2017, cultivated, *W. Sueksakit M10-3* (BK)]; Bangkok [locality no specified, fl., Feb 1869, [as *Garcinia* sp.], cultivated, *C. A. Feilberg s.n.* (C); locality no specified, fl., 14 Mar 1920, cultivated, *A. F. G. Kerr s.n.* (BM); locality no specified, fl., 14 Mar 1920, cultivated, *A. Marcan 102A* (BM); Khong San District, young fr., 20 Feb 1970, cultivated, *J. F. Maxwell 70-23* (BK, L [L2416561])]; **South-Eastern**: Rayong [locality not specified, fr., 16 Jul 2013, cultivated, *Naiyana 01* (BKF)]; Chanthaburi [Khitchakut District, fr., 12 Jul 2003, cultivated, *P. Palee s.n.* (CMUB)]; Trat [Salak Phet Waterfall, Mu Ko Chang National Park, young fr., 29 Mar 2000, cultivated, *T. Wongprasert s.n.* (BKF128511); Wang Saem Subdistrict, Makham District, female fl., 20 Jan 2024, cultivated, *C. Ngernsaengsaruay* et al. *G55-20012024* (BKF)]; **Peninsular**: Surat Thani [Ko Samui, fr., 31 May 1960, cultivated, *Chirayupin 111* (BK); Ban Song Subdistrict, fr., 12 Aug 1979, cultivated, *Supatra 27* (PSU)]; Phangnga [Si Phang Nga National Park, sterile, 17 Dec 2003, cultivated, *A. Sloth 561* (AAU); Ko Phra Thong, fl., 8 Feb 2005, cultivated, *C. Phengklai* et al. *13945* (BKF); Bang Nai Si Subdistrict, Takua Pa District, sterile, 1 Sep 2016, cultivated, *W. Sueksakit M10-1* (BK)]; Nakhon Si Thammarat [locality no specified, sterile, 24 Aug 1980, cultivated, *Students s.n.* (PSU); locality no specified, fr., 20 Aug 1981, cultivated, *Mai 4* (PSU); Khao Luang, fl., 25 Apr 1990, cultivated, *R. Pooma P37* (BKF)]; Phatthalung [Khao Pu-Khao Ya National Park, Si Banphot District, fr., 26 Jul 1986, cultivated, *J. F. Maxwell 86-499* (AAU, BKF, L [L2416615], P [P05061446], PSU)]; Narathiwat [Chat Warin Waterfall, fr., 15 Aug 1995, cultivated, *K. Larsen* et al. *45615* (AAU, BKF)].

### ﻿Excluded and unplaced species

#### ﻿ Garcinia anomala

Taxon classificationPlantaeMalpighialesClusiaceae

Planch. & Triana, Ann. Sci. Nat., Bot., sér. 4, 14: 329. 1860; Laness., Mém. Gen. Garc.: 30. 1872; T. Anderson in Hook. f., Fl. Brit. India 1(2): 266. 1874; Kurz, J. Asiat. Soc. Bengal, Pt. 2, Nat. Hist. 43(2): 87. 1874 et Forest Fl. Burma 1: 89. 1877; Vesque, Epharmosis 2: 17. t. 105. 1889 et in A. DC. & C. DC., Monogr. Phan. 8: 369. 1893; Engl. in Engl. & Prantl, Die Naturlichen Pflanzenfamilien 3(6): 236. 1893; Brandis, Indian Trees: 51. 1906; Kanjilal, P. C. Kanjilal & A. Das, Fl. Assam 1(1): 109. 1934; Maheshw., Bull. Bot. Surv. India 6: 117. t. 1. fig. 7. 1964; S. W. Jones, Morphology and Major Taxonomy of Garcinia (Guttiferae), Ph.D. Thesis (unpublished): 291. 1980; D. G. Long in Grierson & D. G. Long, Fl. Bhutan 1(2): 368. 1984; N. P. Singh in B. D. Sharma & Sanjappa, Fl. Ind. 3: 104. 1993; Wang et al., Phytotaxa 327(2): 167–174. figs 1, 3. 2017.

BFFFF081-3668-563C-AA87-39D9F95B1EBA

Fig. 6

= Garciniapropinqua Craib, Bull. Misc. Inform. Kew 1924(3): 85. 1924; Craib, Fl. Siam. 1(1): 117. 1925; Gagnep. in Gagnep., Fl. Indo-Chine Suppl.: 267. 1943. Type. Thailand, Chiang Mai, Doi Chiang Dao, c. 1500 m alt., fl., 5 Jun 1921, *A. F. G. Kerr 5611* (lectotype, designated by Nazre et al. (2018: 48), K [K000380474!]; isolectotype BM [BM000611616, photo seen].
= Garciniabracteata C. Y. Wu ex Y. H. Li, Acta Phytotax. Sin. 19(4): 490. fig. 1. 1981; H. W. Li et al., Fl. China 13: 44. fig. 31(1–5). 2007. Type. China, Yunnan, Mengla, Mengyuan, 600–700 m alt., male fl., 19 May 1962, *Y. H. Li 4103* (holotype, KUN [KUN0406601, photo seen]).

##### Type.

India, Khasia, 3000–5000 ft alt., female fl., fr., s.d., *J. D. Hooker & T. Thomson 14* (lectotype, designated by Wang et al. (2017: 168), K [K000380443!]; isolectotypes A [reported by Nazre et al. (2018), not seen], BR [BR0000005107803, photo seen], E [E00438018, photo seen], G [G00418238, G00458403, photos seen], L [L0489482, L2409535, U1208227, photos seen], MPU [MPU014371, MPU014372, photos seen], P [P04022020, P04022021, P05062484, P05062485, P05062488, photos seen], W [W0073366, W1889-0318023, W1889-0318024, W1889-0318030, photos seen].

##### Description.

***Habit*** trees, 5–13 m tall, 15–70 cm GBH; latex pale yellow; branches decussate, horizontal or nearly horizontal; branchlets green or yellowish pale green, terete, glabrous. ***Bark*** mottled with dark brown and brown, thin, rather smooth or flaking; inner bark reddish brown. ***Leaves*** decussate; lamina elliptic, oblong-elliptic, ovate or lanceolate-ovate, 6–21.5 × 2.5–9 cm, apex acute, bluntly acute or shortly acuminate, base cuneate or obtuse, sometimes rounded or oblique, margin entire or repand, slightly revolute, coriaceous, shiny dark green above, pale green below, glabrous on both surfaces, midrib flattened above, raised below, secondary veins 11–21 each side, curving towards the margin and connected in distinct loops and united into an intramarginal vein, flattened above, slightly raised below, visible on both surfaces, with intersecondary veins, veinlets reticulate, visible on both surfaces, interrupted long wavy lines of differing lengths, running across the secondary veins to the apex, obscure or visible below; petiole green, 0.4–2 cm long, 1.5–3 mm in diam., grooved above, transversely rugose, glabrous, with a basal appendage clasping the branchlets; young leaves brownish red or reddish brown, turning pale green, glossy. ***Inflorescences*** axillary, cymose, often in a cluster of 3 flowers or 2–7 flowers; leafy bracts 2, opposite, ovate, broadly ovate or lanceolate-ovate, 0.7–4.3 × 0.4–2 cm, apex acute, base obtuse, margin entire, coriaceous; petiole 1–4.5 mm long, 0.5–1.5 mm in diam.; peduncle green, short to slender, 0.2–4 cm long, 1–2.5 mm in diam., glabrous. ***Flowers*** unisexual, plants dioecious, 4-merous, 1–1.5 cm in diam.; bracteoles 2, opposite, caducous, triangular, 1.5–2.5 × 1.3–2 mm; pedicel 3–6 mm long, 0.8–1.5 mm in diam., glabrous; sepals and petals decussate, concave, gradually reflexed after anthesis, glabrous; sepals 4, pale green, orbicular, suborbicular, broadly ovate or lanceolate-ovate, 2–6 × 1.5–4 mm, the outer pair slightly smaller than the inner pair; petals 4, pale yellow or yellowish white, elliptic, oblong-elliptic or broadly elliptic, 4.5–6 × 2.5–4 mm, subequal. ***Flower buds*** subglobose to globose, 4–5 mm in diam. ***Male flowers***: stamens white or creamish white, numerous, united in a central depressed globose bundle surrounding the pistillode; filaments very short; anthers 2-thecous, small, longitudinally dehiscent; pistillode small. ***Female flowers***: staminodes many; filaments short, basally connate into a cup surrounding the base of the ovary but distally free; anthers yellow, small; pistil fungiform, 4–5.5 mm long; ovary pale green, broadly ovoid, 2.5–3.5 × 3–4 mm, unlobed, glabrous, 1–2-locular; stigma yellow, sessile, slightly convex, weakly lobed, 4–5 mm in diam., smooth. ***Fruits*** berries, dark green, turning purple when ripe, smooth with fine longitudinal striate, glabrous, ellipsoid or broadly ellipsoid, 1.8–2.5 × 1.2–2.2 cm, without or with a short, thick beak; persistent stigma dark brown or blackish brown, flattened, weakly lobed, 4.5–6 mm in diam. smooth; persistent sepals green, slightly larger than in flowering materials; fruiting stalk green, 0.6–1 cm long, 1.2–2.5 mm in diam., glabrous. ***Seeds*** 1–2, c. 8 × c. 6 mm. The size of seeds was taken from Vesque (1893).

**Figure 6.**
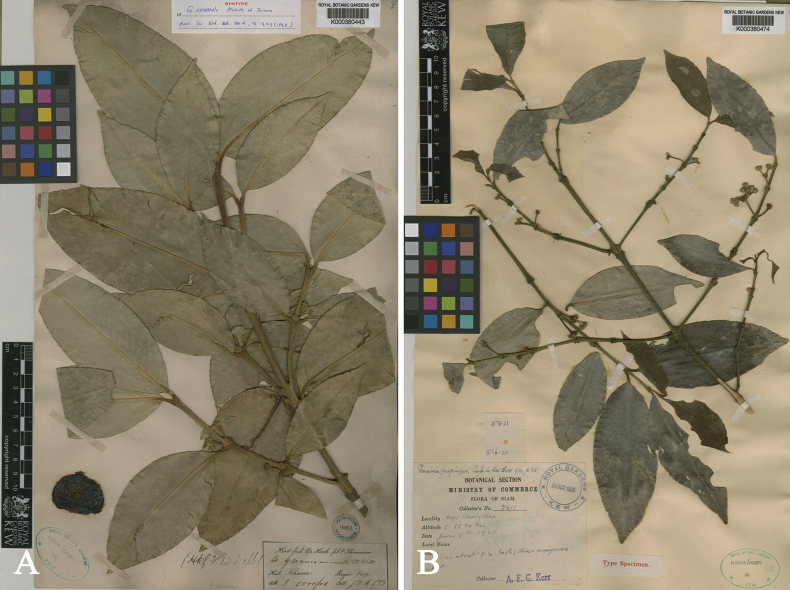
*Garciniaanomala***A** lectotype of *Garciniaanomala*, *J. D. Hooker & T. Thomson 14* (K [K000380443]) from Khasia, India, designated by Wang et al. (2017)**B** lectotype of *Garciniapropinqua*, a synonym of *Garciniaanomala*, *A. F. G. Kerr 5611* (K [K000380474]) from Doi Chiang Dao, Chiang Mai Province, Thailand, designated by Nazre et al. (2018). Photos: Royal Botanic Gardens, Kew, England. https://powo.science.kew.org/taxon/urn:lsid:ipni.org:names:427802-1.

##### Distribution.

India [Eastern India (Sikkim, Assam, Meghalaya), Bangladesh, Myanmar (Martaban), China (South Guangxi, South and South-East Yunnan), Vietnam, Thailand.

##### Distribution in Thailand.

**Northern**: Chiang Mai, Chiang Rai, Tak.

##### Habitat and ecology.

It is found in lower montane rain forests or on limestones in lower montane rain forests, at elevations of 1,300–1,750 m amsl.

##### Phenology.

Flowering February to October; fruiting September to November.

##### Conservation status.

*Garciniaanomala* is widely distributed from Eastern India to North Indo-China and Thailand. It is known from many localities and has a large Extent of Occurrence (EOO) of 1,980,330.45 km^2^ and a relatively large Area of Occupancy (AOO) of 156 km^2^. In Thailand, this species is known to be naturally distributed in three provinces of the northern region, and has an EOO of 26,685.60 km^2^ and an AOO of 48 km^2^. Because of this wide distribution and the number of localities, it is considered LC.

##### Etymology.

The specific epithet of *Garciniaanomala* is a Latin word meaning abnormal (Stearn 1992), unlike its allies, out of the ordinary (Gledhill 2002) and refers to inflorescences subtended by 2 small leaf-like bracts which can be used as a spot character for distinguishing the species. The specific epithet of *G.propinqua* is a Latin word meaning closely allied, of near relationship, related (Stearn 1992; Gledhill 2002), in reference to *G.propinqua* being closely related to *G.anomala*. The specific epithet *Garciniabracteata* is a Latin word meaning with bracts, bracteate (Stearn 1992; Gledhill 2002) and refers to bracteate cymes (inflorescences with 2 opposite leafy bracts).

##### Vernacular names.

**Phawa thiam bai pradap** (พะวาเทียมใบประดับ) (suggested here); Dieng-sa-slung, Dieng-soh-lang-sain (Jain, India), Dieng-soh-kwang, Soh-lain-khlaw (Khasi, India); Haibung (Manipur, India); Thechu (Garo, India).

##### Uses.

The ripe fruits have a sour taste (from the specimen *B. Hansen & T. Smitinand 12915*).

##### Notes.

Based on morphological characters and molecular data Nazre et al. (2018) excluded several species that were included in GarciniasectionGarcinia by Jones (1980). *Garciniaanomala* is treated by Jones (1980) as belonging to GarciniasectionGarcinia but Nazre et al. (2018) exclude it from the section. Molecular results of Gaudeul et al. (2024) fully support the decision of Nazre et al. (2018); they recovered two major lineages, nine major clades, and 11 sections. *G.anomala* is unplaced species within these11 sections (Gaudeul et al. 2024). It differs from GarciniasectionGarcinia by having axillary cymose inflorescences often in clusters of 3 flowers or 2–7 flowers on the short to slender peduncles, each subtended by 2 small leaf-like bracts and the male flowers having glomerate, depressed globose bundles of stamens surrounding the pistillode. From our examination of specimens, we agree with the results of Nazre et al. (2018).

Wang et al. (2017) treated *Garciniabracteata* and *G.propinqua* as synonyms of *G.anomala*. *G.anomala* is the earliest named species and thus has nomenclatural priority. The three purported species of *Garcinia* have similar morphological characters (overlapping variation in leaf shape and size, petiole length, foliar bract shape and peduncle length). There were no significant differences between these traits. They asserted that the traits previously used for distinguishing between *G.bracteata*, *G.anomala*, and *G.propinqua* are unreliable for distinguishing these species.

According to Singh (1993) the shape, size, and color of fruits are ellipsoid, c. 4.2 × c. 3.5 cm, and dark olive green, turning orange-yellow when ripe; however, from our examinations, we found the fruits are ellipsoid or broadly ellipsoid, 1.8–2.5 × 1.2–2.2 cm, without or with a short, thick beak, dark green, and turning purple when ripe (color of ripe fruits from the specimen *B. Hansen & T. Smitinand 12915*).

*Garciniaanomala* was described by Planchon and Triana (1860: 329), who cited the specimens collected by *W. Griffith* (without collector number) and *J. D. Hooker & T. Thomson 14* in Khasia, India. The name *G.anomala* has been lectotypified thrice, firstly, Wang et al. (2017: 168) lectotypified this name using the specimen *J. D. Hooker & T. Thomson 14* at K [K000380443], secondly, Nazre et al. (2018: 47) lectotypified this name using the same collector number at MPU [without barcode] with isolectotypes at A, E, K, and L [without barcodes], and thirdly, Shameer and Mohanan (2019: 181) selected the specimen *W. Griffith 848* at G [G00458432] as the lectotype, with isolectotypes at CAL [CAL0000046566], and W [W0073367]. Therefore, the first lectotypification has priority. We located the lectotype at K [K000380443] with isolectotypes at BR [BR0000005107803], E [E00438018], G [G00418238, G00458403], L [L0489482, L2409535, U1208227], MPU [MPU014371, MPU014372], P [P04022020, P04022021, P05062484, P05062485, P05062488], US [US02961086], and W [W0073366, W1889-0318023, W1889-0318024, W1889-0318030], but we could not find an isolectotype at A.

*Garciniapropinqua* was described by Craib (1924: 85), who cited the specimen *A. F. G. Kerr 5611* collected from Doi Chiang Dao, Chiang Mai Province, Thailand but he did not mention the name of the herbaria where the materials were housed, and following Art. 9.6 of the ICN (Turland et al. 2018), they constitute syntypes. Nazre et al. (2018) selected this specimen at K [K000380474] as the lectotype, with an isolectotype at BM [BM000611616].

*Garciniabracteata* was named by C. Y. Wu but unpublished, and then this name was described by Li (1981: 490), who cited the specimen *Y. H. Li 4103* collected from Mengyuan, Mengla, Yunnan, China, and housed in KUN as the holotype.

##### Additional specimens examined.

**Thailand. Northern.** Chiang Mai [Doi Chiang Dao, fl., 28 Oct 1979 [as *Garcinia* sp.], *T. Shimizu* et al. *T-20980* (AAU, BKF); Doi Chiang Dao Wildlife Sanctuary, Chiang Dao District, fr., 9 Nov 1995 [as *G.propinqua*], *J. F. Maxwell 95-1129* (CMUB); Doi Ang Khang, Fang District, fl., 27 May 1998 (as *G.bracteata*), *T. Wongprasert* et al. *s.n.* (BKF124408); Doi Ang Khang, fl., s.d. [as *Garcinia* sp.], *P. Triboun s.n.* (BK265941)]; Chiang Rai [Summit of Doi Tung near temple, Mae Sai District (originally “Mae Fa Luang District” on the label), fr., 11 Oct 1997 [as *Garcinia* sp.], *R. Pooma & M. Tamura RP-MT10* (BKF, K); Doi Tung, near Wat Phra That Doi Tung, Huai Khrai Subdistrict, Mae Sai District, male fl., 23 May 2006 [as *G.propinqua*], *J. F. Maxwell 06-312* (CMUB, L [L3878616], QBG); ibid., fr., 6 Sep 2006 [as *G.propinqua*], *J. F. Maxwell 06-637* (CMUB, L [L3812984], QBG); along trail near summit of Pha Hung, above Wat Phra That Doi Tung, fr., 22 Oct 2012 [as *G.propinqua*], *M. van de Bult 1275* (BKF, CMUB, L [L4311877]); near Wat Phra That Doi Tung, Mae Sai District (originally “Mae Fa Luang District” on the label), fl., 27 Feb 2003 [as *Garcinia* sp.], *R. Pooma & V. Chaemchumroon 3742* (BKF, SING [SING0095616]); near Wat Phra That Doi Tung, Mae Sai District, male fl., 19 May 2020 [as *G.propinqua*], *M. van de Bult 1732* (BKF); Phu Chi Fa, male fl., 20 Mar 2000 [as *Garcinia* sp.], *BKF Sc404* (BKF180336)]; Tak [Doi Pae Poe, about 90 km NW of Tak, female fl., 14 Mar 1968 [as G.cf.anomala], *B. Hansen & T. Smitinand 12915* (BKF, AAU, C, K, L [L2408816], P [P00329869])].

**India.** Khasia, fl., s.d., *J. D. Hooker & T. Thomson s.n.* (P [P05062486]); Indes Orientales, locality not specified, female fl., fr., 1859, *J. D. Hooker & T. Thomson s.n.* (P [P05062491]); East Bengal, Khasya, male fl., s.d., distributed at the Royal Botanic Gardens, Kew (1861–1862), *W. Griffith 848* (CAL [CAL0000046566], G [G00458432], K [K000677605, K000677606], L [L0489483], P [P00329868]), US [US02961086], W [W0073367]); Khasia, fl. s.d., *W. Griffith 654* (K [K000677607]); locality not specified, fl., 1843, *W. Griffith s.n.* (P [P05062487]); Khasia, fr., 1864, *Unreadable s.n.* (P [P05062482]); India, Jaintia (originally “Jaintea” on the label), 14 Dec 1885, *C. B. Clarke 42547H* (G [G00458506]), *C. B. Clarke 42547J* (US [US02961087]); Assam, male fl., 1893, *G. King’s Collector s.n.* (L [L2408817], P [P05062492], US [US02961088]); Assam, Laitlynkot, Khasi Hills, young fr., 13 Jul 1949, *T. R. Chand 1786* (L [L2409534]); Assam, Cherrapunjee, Khasi Hills, 4000 ft alt., fl., 21 Jul 1952, *W. N. Koelz 30721* (L [L2409592]); ibid., fl., 29 Apr 1952, *W. N. Koelz 29534a* (L [L2409594]; ibid., fl. 9 May 1952, *W. N. Koelz 29795* (L [L2409595]).

**China.** Yunnan, Mengyuan, Mengla, Xishuangbanna, 850 m alt., fl., 4 Sep 2004 [as *G.bracteata*], Z*hou Shi-shun 2056* (QBG); Guangxi, Na Po County, Nong Hua, fl., 5 Jun 1989 [as *G.bracteata*], *H. Q. Wen W014* ([US02961079]).

**Vietnam.** Ha Giang, Dong Van District, Municipality Ho Quang Phin, Vicinity of Ta Xa Village, male fl., 28 Apr 1999 [as *Garcinia* sp.], *P. K. Loc* et al. *CBL1740* ([P05061735]); Ha Giang, Meo Vac District, Municipality Sung Chang, Vicinity of Lu Lu Phin Village, Cao Bang Limestone, male fl., 29 Apr 1999 [as *Garcinia* sp.], *P. K. Loc* et al. *CBL1851* (P [P05061727]); Cao Bang, Nguyen Binh District, Municipality Ca Thanh, Cao Bang Limestone, male fl., 13 Apr 1999 [as *Garcinia* sp.], *P. K. Loc* et al. *CBL1317* (P [P05061730]); Hoa Binh Province, Mai Chau District, Hang Kla, fr., 22 Sep 2005 [as *Garcinia* sp.], *Vu Xuan Phong* et al. *HNK750* (K [K000576423]).

## Supplementary Material

XML Treatment for Garcinia L. section Garcinia

XML Treatment for Garcinia celebica

XML Treatment for Garcinia exigua

XML Treatment for Garcinia mangostana

XML Treatment for Garcinia anomala

## ﻿Additional information

### Author ORCIDs

Chatchai Ngernsaengsaruay https://orcid.org/0000-0002-7131-976X

Pichet Chanton https://orcid.org/0009-0001-7325-6109

Minta Chaiprasongsuk https://orcid.org/0009-0006-9146-1161

### Data availability

All of the data that support the findings of this study are available in the main text.
